# Combination Treatment Targeting mTOR and MAPK Pathways Has Synergistic Activity in Multiple Myeloma

**DOI:** 10.3390/cancers15082373

**Published:** 2023-04-19

**Authors:** Kaiyan Sun, Ling Jin, Jana Karolová, Jan Vorwerk, Stephan Hailfinger, Bertram Opalka, Myroslav Zapukhlyak, Georg Lenz, Cyrus Khandanpour

**Affiliations:** 1 Department of Medicine A, Hematology, Hemostaseology, Oncology and Pneumology, University Hospital Münster, 48149 Münster, Germany; 2 Institute of Pathological Physiology, First Faculty of Medicine, Charles University, 12108 Prague, Czech Republic; 3 Department of Hematology and Stem Cell Transplantation, West German Cancer Center (WTZ), University Hospital Essen, 45147 Essen, Germany; 4 Department of Hematology and Oncology, University Hospital Schleswig-Holstein and University of Lübeck, 23538 Lübeck, Germany

**Keywords:** multiple myeloma, mTOR, temsirolimus, MEK, trametinib, targeted therapy

## Abstract

**Simple Summary:**

Multiple myeloma (MM) is characterized by the clonal accumulation of abnormal plasma cells in the bone marrow. Although current treatments have improved the survival rates of patients with MM, MM remains incurable for most patients due to refractory disease and relapse. Thus, new therapeutic strategies are needed. Constitutive activation of the PI3K/AKT/mTOR signaling pathway has been identified in MM, leading to uncontrolled tumor growth and survival. In this study, we revealed that an inhibitor targeting mTOR with temsirolimus exhibited different anti-proliferation activity across our MM cell lines, regardless of their genetic features. Combination treatment of the mTOR inhibitor with a MEK inhibitor led to a synergistic anti-proliferation effect in MM cell lines. Thus, our study provides a rationale for future clinical trials of temsirolimus in MM patients as a component of combination therapy and suggests that MM cases with a high p-S6 but low p-AKT level could benefit from this combination. This was irrespective of cytogenetic features and reveals an additional way to classify MM cells.

**Abstract:**

Multiple myeloma (MM) is an incurable, malignant B cell disorder characterized by frequent relapses and a poor prognosis. Thus, new therapeutic approaches are warranted. The phosphatidylinositol-3-kinase (PI3K) pathway plays a key role in many critical cellular processes, including cell proliferation and survival. Activated PI3K/AKT (protein kinases B)/mTOR (mammalian target of rapamycin) signaling has been identified in MM primary patient samples and cell lines. In this study, the efficacy of PI3K and mTOR inhibitors in various MM cell lines representing three different prognostic subtypes was tested. Whereas MM cell lines were rather resistant to PI3K inhibition, treatment with the mTOR inhibitor temsirolimus decreases the phosphorylation of key molecules in the PI3K pathway in MM cell lines, leading to G_0_/G_1_ cell cycle arrest and thus reduced proliferation. Strikingly, the efficacy of temsirolimus was amplified by combining the treatment with the Mitogen-activated protein kinase kinase (MEK) inhibitor trametinib. Our findings provide a scientific rationale for the simultaneous inhibition of mTOR and MEK as a novel strategy for the treatment of MM.

## 1. Introduction

Multiple myeloma (MM) is characterized by the clonal accumulation of abnormal plasma cells in the bone marrow (BM). It is the second most common hematological malignancy, accounting for approximately 14% of all hematological cancers in Europe and North America [1]. Therapies against MM have significantly improved over the past decades, and many novel approaches, including hematopoietic stem cell transplantation, novel agents such as bortezomib, chimeric antigen receptor T-cell immunotherapy, and immunomodulatory drugs such as lenalidomide, have been incorporated into the therapeutic algorithms [2,3,4,5]. Although these treatments improved the rate of complete response and survival rates of patients with MM, MM remains incurable for most patients due to the high relapse frequency and increasing resistance driven by the accumulation of genetic alterations [6]. In addition, targeted drugs successfully used in other B-cell malignancies, such as rituximab and obinutuzumab, exhibit poor efficacy in MM [7,8]. Therefore, new therapeutic strategies are urgently required.

Previous studies have revealed that the phosphatidylinositol-3-kinase (PI3K)/protein kinases B (AKT)/mammalian target of rapamycin (mTOR) pathway plays a key role in various cellular processes, including survival, migration, and cell proliferation [9,10]. Inhibitors targeting PI3-kinase, the upstream activator of the PI3K axis, rose as a promising target for this reason. Previously, PI3K inhibitors studied in a variety of B-cell malignancies, such as diffuse large B-cell lymphoma and indolent lymphoma, displayed novel effects [11,12]. On the contrary, PI3K inhibitors studied in MM were limited. Hence, to address this point, additional studies focused on this pathway are highly warranted in MM. In addition, inhibitors targeting AKT, the central node in the PI3K axis, were also evaluated in lymphoma but rarely reported in MM [13].

As a crucial downstream effector of the PI3K pathway, mTOR exists in two different forms: mTOR complex 1 (mTORC1) and mTORC2, which have distinct functions [14]. Dysregulation of the mTOR signaling cascade has been identified in numerous tumors, including MM, leading to uncontrolled tumor growth and survival [15,16,17]. Therefore, mTOR could serve as one of the rational targets in MM therapy. mTORC1 is sensitive to rapamycin and activated mTORC1 could phosphorylate S6 and 4E-BP1, eventually regulating transcription, ribosomal biogenesis, and protein translation [18,19]. Temsirolimus, a rapamycin analog that was approved by the FDA in 2007, is the first-line drug used in renal cell carcinoma [20]. Temsirolimus was also evaluated in a phase II trial of MM and showed modest activity as a single agent [21]. Therefore, our interest was to investigate whether targeting additional pathways or drug combinations could help to improve the efficacy of temsirolimus. From one preclinical study, the combination of lenalidomide with rapamycin has displayed synergy and represents a promising combination in MM [22].

Another rational target would be the mitogen-activated protein kinase (MAPK)/extracellular signal-regulated kinase (ERK) pathway, as previous studies showed that mutations in this pathway are one of the most common mutations presented in MM patients [23]. This signaling pathway is composed of several protein kinases, including RAS, RAF, Mitogen-activated protein kinase kinase (MEK), and ERK, and is active downstream of important growth factor receptors [24,25]. In this signaling cascade, RAS acts upstream, RAF acts as MAP3K, MEK acts as MAPKK, and MAPK/ERK is the unique substrate of MEK [25]. Activated ERK phosphorylates multiple targets into the nucleus to phosphorylate a broad variety of transcription factors, thus driving cell growth, development, differentiation, and mobility [26]. In many malignancies, the MAPK/ERK pathway is frequently dysregulated and is therefore an interesting target for cancer therapy [25,27]. Trametinib, an oral MEK1 and MEK2 inhibitor, has been approved by the FDA in 2014 for BRAF V600 mutated melanoma treatment in a combination with the BRAF inhibitor dabrafenib. The common side effects include rash, peripheral edema, and nausea [28].

In this study, we discovered the anti-MM activity of the mTOR inhibitor temsirolimus in a panel of MM models, representing three prognostic subtypes. Based on molecular analysis, we identified that the combination with the MEK1/2 inhibitor trametinib enhanced the efficacy of temsirolimus in various MM cell lines.

## 2. Materials and Methods

### 2.1. Cell Lines and Cell Cultures

MM.1S, AMO-1, and U266 human MM cell lines were kindly provided by Stephan Mathas (Charite-Universitätsmedizin Berlin, Berlin, Germany). LP-1, SKMM-2, OPM-2, RPMI-8226, and IM-9 human MM cell lines were kindly provided by Michael Hummel (Charite-Universitätsmedizin Berlin, Berlin, Germany). OH-2, KJON, and Volin human MM cell lines were kindly provided by Thea Kristin Våtsveen (Norwegian University of Science and Technology, Trondheim, Norway). MM.1S, LP-1, SKMM-2, OPM-2, AMO-1, IM-9, and RPMI-8226 cell lines were cultured in RPMI-1640 medium (Cat.#21870076, Gibco^®^, Thermo Fisher Scientific, Waltham, MA, USA) with 20% or 10% of fetal calf serum (PAN-Biotech, Aidenbach, Germany), and Penicillin-Streptomycin (Cat.#P30-3302, PAN-Biotech). U266 and Volin cell lines were cultured in IMDM medium (Cat.#1023936, Gibco^®^, Thermo Fisher Scientific) with 20% human plasma (Blood bank of University Hospital Münster, Münster, Germany), Penicillin-Streptomycin, Heparin (Cat.#H3393-100KU, Sigma-Aldrich, Saint Louis, MO, USA), and 2-sulfanylethanol (Cat.#P70-05020, PAN-Biotech). OH-2 and KJON cell lines were cultured in IMDM medium with 20% human plasma, Penicillin-Streptomycin, Heparin, 2-sulfanylethanol, and human IL-6 (Cat.#200-06, Peprotech, Rocky Hill, NJ, USA).

### 2.2. Chemicals

Trametinib (Cat.#S2673), alpelisib (Cat.#S2814), pictilisib (Cat.#S1605), AZD8186 (Cat.#S7694), capivasertib (Cat.#S8019), AZD8835 (Cat.#S7966), eganelisib (Cat.#S8330), ferrostatin-1 (Cat.# S7243), and idelalisib (Cat.#S2226) were purchased from Selleckchem (Houston, TX, USA). TGX-221 (Cat.#S528113) was purchased from Merck Millipore (Burlington, MA, USA). Temsirolimus (Cat.#PZ0020) was purchased from Sigma-Aldrich. The inhibitors mentioned above were dissolved in DMSO (trametinib, alpelisib, pictilisib, AZD8835, eganelisib, idelalisib, AZD8186, capivasertib, TGX-221, and temsirolimus) and stored at −20 °C.

### 2.3. Cell Viability Assay

MM cell lines were seeded in 96-well plates with a density of 5 × 10^4^ cells/mL in a final volume of 100 µL and incubated with different concentrations of selected drugs or corresponding concentrations of DMSO dissolved in the medium, as mentioned above. After 5 d of incubation at 37 °C, the cell suspensions were added to the equal volume of CellTiter-Glo^®^ Reagent (Cat.#G7573, Promega, Madison, WI, USA) in new 96-well plates. Cell viability was measured using a Victor^®^ multilabel plate reader (PerkinElmer, Waltham, MA, USA) after the suspensions were mixed with CTG reagent for 30 min on an orbital shaker at room temperature (RT).

### 2.4. Western Blotting

Western blotting was performed as previously described [11]. The amount of protein in the cell lines was quantified by BCA reagent (Cat.#23227, Thermo Fisher Scientific). Protein lysates were extracted from the MM cell lines after incubation with different drugs or corresponding amounts of DMSO. Proteins present in lysates were separated by sodium dodecyl sulfate-polyacrylamide gel electrophoresis on 8–12% acrylamide gels and transferred to Immobilon^®^-E polyvinylidene membranes (Cat.#IEVH00005, Merck Millipore), which were performed at 100 V for 90 min. Primary antibodies used for Western Blotting were obtained from Cell Signaling Technology (Danvers, MA, USA): PI3Kα (Cat#.4249S), PI3Kβ (Cat.#3011S), PI3K γ (Cat.#5405S), panAKT (Cat.#9272), p-AKT S473 (Cat.#4058), p-AKT T308 (Cat.#2965), p-p70S6K (Cat.#9234), p-S6 (Cat.#2211), p-4E-BP1 (Cat.#2855), p-ERK (Cat.#4370), cyclin D1 (Cat.#55506), CDK4 (Cat.#12790), CDK6 (Cat.#13331), LC3B (Cat.#3868), Merck Millipore: PI3Kδ (Cat.#04-401), Santa Cruz Biotechnology (Dallas, TX, USA): cyclin A (Cat.#sc-59645), PTEN (Cat.#sc-7974), Thermo Fisher Scientific: p-PRAS40 (Cat.#44-1100G), and Sigma-Aldrich: Tubulin (Cat.#T9026), β-actin (Cat.#A5441). The primary antibodies mentioned above were diluted as per manufacturer’s instructions.

### 2.5. Apoptosis Assay

For each sample, 0.5–1 × 10^6^ cells were harvested by centrifugation at 1200 rpm for 5 min at RT after 48 h of inhibitor treatment. After washing once with PBS, cell pellets were resuspended in 100 μL of binding buffer (Cat.#556454, Thermo Fisher Scientific) with 0.5 μL Annexin V APC (Cat.#640941, BioLegend, San Diego, CA, USA) and incubated for 10 min in the dark at RT. Subsequently, 100 μL of binding buffer (Thermo Fisher Scientific) containing 0.5 μL PI (Cat.#P3566, Thermo Fisher Scientific) was added and incubated again for 10 min at 4 °C. Analysis was performed with Attune^®^ NxT acoustic focusing cytometry (Thermo Fisher Scientific).

### 2.6. Cell Cycle Analysis

The cell cycle assay was performed using a two-step cell cycle analysis Kit (ChemoMetec, Allerod, Denmark), according to the manufacturer’s instructions. Briefly, the cell lines mentioned above were seeded in 6-well plates at a density of 0.5 × 10^6^/mL. Cell lines were incubated with selected drugs or corresponding concentrations of DMSO dissolved in the medium, as previously mentioned. Next, 0.5–1 × 10^6^ cells were harvested by centrifuging for 5 min at RT after 24 h incubation at 37 °C. After washing, PBS was thoroughly removed. Pellets were resuspended in lysis buffer (Cat.#910-3010) containing 500 μg/mL DAPI (Cat.#910-3012) with a final concentration of 10 μg/mL DAPI and incubated at 37 °C for 5 min. 250 μL of stabilization buffer (Cat.#910-3011) was added after the incubation, and cell suspensions were measured by NucleoCounter using NC-Slide A8^®^ (Cat.#942-0003) (10 μL). Data were analyzed using ModFit LT version 5.0 (Verity Software House, Topsham, ME, USA).

### 2.7. CFSE Proliferation Staining

The determination of proliferation using carboxyfluorescein diacetate succinimidyl ester (CFSE) staining (Cat.#C34570, eBioscience, San Diego, CA, USA) was performed as previously described [11]. The proliferation was quantified by mean fluorescence intensity (MFI) 5 days post incubation with the inhibitor or corresponding concentrations of DMSO at 37 °C.

### 2.8. Calculation of Combination Effect

The combination was tested at different concentrations, in which each inhibitor was added as a single agent or in combination. For synergistic effects, all dose combinations were evaluated with the measurement of cell viability and analyzed based on the Loewe model. Loewe synergy scores larger than 10, from −10 to 10, and less than 10 correspond to synergistic, additive, and antagonistic, respectively [29,30]. Synergy scores were determined using Combenefit (Slashdot Media, San Diego, CA, USA) [31].

### 2.9. Statistical Analysis

All data are shown as the mean ± standard deviations (SD) from at least three independent experiments. Statistical analysis was carried out by GraphPad Prism version 6.0 (GraphPad Software, San Diego, CA, USA). Two sample comparisons were evaluated using a student’s *t*-test, and multiple comparisons were analyzed by one-way analysis of variance (ANOVA). *p* < 0.05 was considered as significant (* *p* ≤ 0.05, ** *p* ≤ 0.01, *** *p* ≤ 0.001, **** *p* ≤ 0.0001).

## 3. Results

### 3.1. MM Cell Lines Show Various Sensitivities to PI3K and mTOR Inhibitors

**Figure 1:**
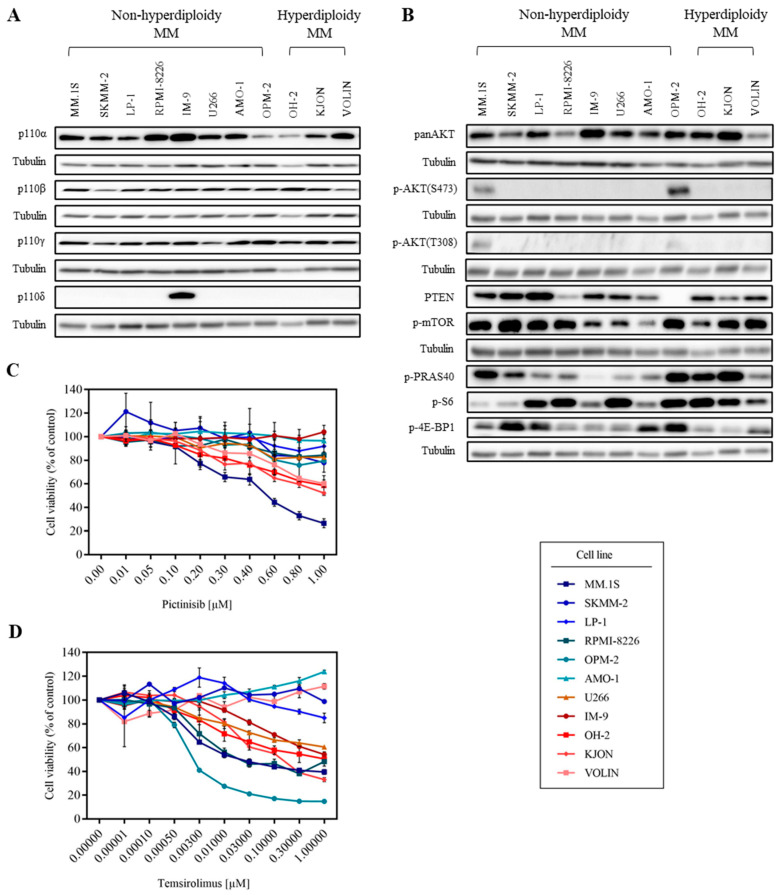
Different sensitivities of MM cell lines to pan PI3K and mTOR inhibitors. (**A**) Basal levels of PI3K isoforms a, β, γ, and δ in MM cell lines were detected by Western blotting, using isoform-specific antibodies. Tubulin was the loading control. Representative results from at least three independent experiments are shown. (**B**) Basal levels of AKT (S473 and T308), mTOR, PRAS40, S6, and 4E-BP1 phosphorylation, and panAKT and PTEN in MM cell lines were detected by Western blotting using specific antibodies. Tubulin was the loading control. Representative results from at least three independent experiments are shown. (**C**) MM cell lines were incubated for 5 days with the pan-class I PI3K inhibitor pictinisib. Viable cells were determined. Representative results from at least three independent experiments are shown. Error bars indicate standard deviations. (**D**) MM cell lines were incubated for 5 days with the mTOR inhibitor temsirolimus. Viable cells were determined. Representative results from at least three independent experiments are shown. Error bars indicate standard deviations.

To better understand why PI3K inhibitors have exhibited promising effects in lymphoma but were studied in MM limitedly, more investigations on the PI3K pathway in MM is needed. Thus, to determine the activation status of the PI3K pathway in MM, we first prepared cell lysates from eleven MM cell lines and checked the basal expression levels of the PI3K isoforms and downstream molecules by Western blotting. Molecular characteristics for these eleven cell lines are summarized in Table S1 [32,33,34,35]. The expression of the catalytic PI3K isoforms α, β, and γ was observed virtually in all the tested cell lines, although basal levels varied. The δ isoform was only detectable in IM-9 cell lines (Figure 1A and Figure S5). We also checked the basal expression levels of PI3K pathway key molecules such as AKT and Phosphatase and Tensin homolog (PTEN) as well as phosphorylation of AKT (S473 and T308), mTOR, PRAS40, S6, and 4E-BP1. We found that p-AKT was only detectable in MM.1S and OPM-2 cell lines, whereas the suppressor of the PI3K pathway, PTEN, was only lost in the OPM-2 cell line. However, interestingly, the phosphorylation of mTOR, PRAS40, S6, and 4E-BP1 was observed in most MM cell lines (Figure 1B and Figure S6).

Next, we performed a pharmacologic screen using different inhibitors against PI3K, AKT, and mTOR by determining cell viability after treatment of cultures with respective inhibitors for 5 d [33,36]. MM.1S, a cell line with detectable p-AKT and representing MM with a poor prognosis, was the only MM cell line sensitive to the AKT inhibitor capivasertib [37] (IC_50_ = 0.058 μM), and some of the screened PI3K inhibitors, such as the pan-class I PI3K inhibitor pictilisib [38] (IC_50_ = 0.51 μM) (Figure 1C and Figure S1A,B). Alpelisib [39], TGX-221 [40], eganelisib [41], idelalisib [42], and AZD8186 [43], inhibitors against PI3Kα, β, γ, δ, or β/δ, respectively, induced no toxicity in these MM models (Figure S1C–G), suggesting that targeting PI3K is not effective in our MM cell lines. Temsirolimus, which is effective in various tumors according to previous studies [44,45], showed toxicity in seven of eleven MM cell lines (Figure 1D). OPM-2, a cell line representing poor prognosis, showed high sensitivity to temsirolimus (IC_50_ = 0.0031 μM). Four cell lines, MM.1S, RPMI-8226, U266, and IM-9, are also known to mirror poor prognosis, and two cell lines, OH-2 and KJON, representing favorable prognosis, showed moderate sensitivities to temsirolimus (IC_50_ = 0.055 μM, 0.081 μM, 2.38 μM, 1.03 μM, 0.45 μM, and 0.15 μM respectively). From the IC_50_ values, we did not observe distinct responses of the eleven MM cell lines representing three prognostic subtypes to these inhibitors (Table S2). For further experiments, we classified the eleven MM cell lines into two groups, sensitive and resistant, based on their IC_50_ values against temsirolimus or pictinisib.

### 3.2. mTOR Inhibition Downregulates PI3K Pathway Key Molecules

**Figure 2:**
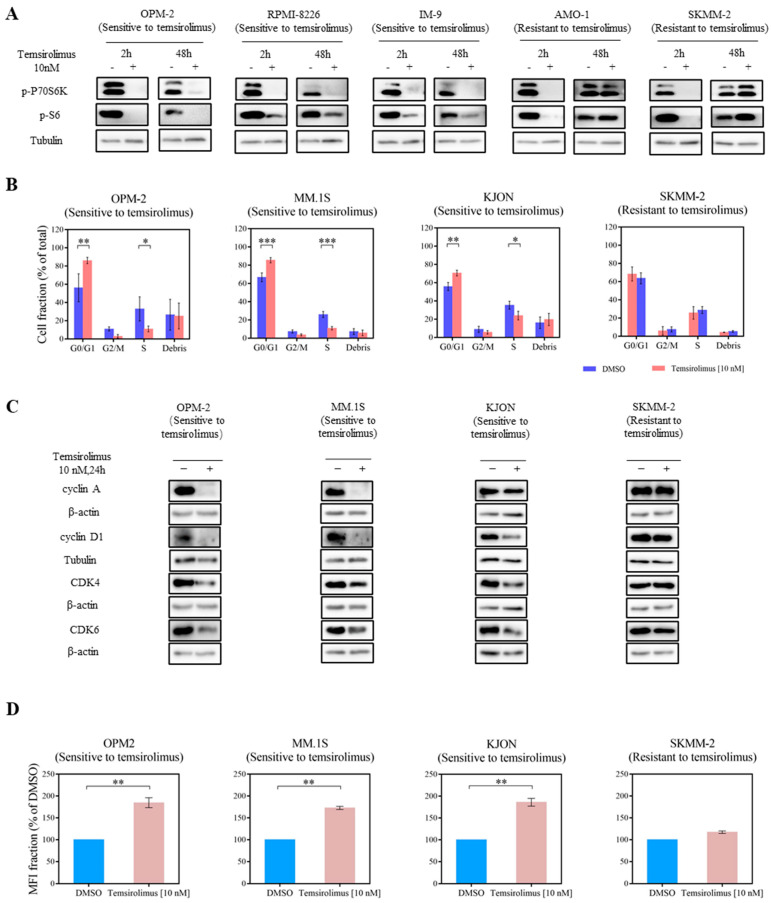
Temsirolimus is active in vitro. (**A**) Treatment with 10 nM of temsirolimus for 2 h resulted in decreased phosphorylation of P70S6K and S6 in OPM-2, RPMI-8226, IM-9, AMO-1, and SKMM-2 cell lines. A restoration of P70S6K and S6 phosphorylation was observed in AMO-1 and SKMM-2 cell lines after treatment with 10 nM temsirolimus for 48 h. Tubulin was the loading control. These results are representative of three separate experiments. (**B**) Temsirolimus treatment induced cell cycle arrest. 24 h of inhibitor treatment resulted in an increase of G_0_/G_1_ stage in OPM-2, MM.1S, and KJON cell lines, whereas no cell cycle arrest was detectable in the SKMM-2 cell line. Data are expressed as the means ± standard deviation of at least 3 independent experiments. Error bars indicate standard deviations. * *p* ≤ 0.05, ** *p* ≤ 0.01, *** *p* ≤ 0.001. (**C**) Treatment with 10 nM temsirolimus for 24 h resulted in decreased cyclin D1, CDK4, and CDK6 in OPM-2, KJON, and MM.1S cell lines and decreased cyclin A in OPM-2 and MM.1S cell lines. No downregulation could be observed in the SKMM-2 cell line. Tubulin and β-actin were the loading controls. These results are representative of three separate experiments. (**D**) Temsirolimus treatment reduced cell proliferation. CFSE dilutions were measured 5 days after treatment with temsirolimus. Cell proliferation was measured by MFI fraction quantification. In OPM-2, MM.1S, and KJON cell lines, proliferation was decreased by temsirolimus. Representative results of three independent experiments are shown. Error bars indicate standard deviations. ** *p* ≤ 0.01.

Since PI3K signaling was very heterogeneous in the tested MM cell lines, and the PI3K and mTOR inhibitors displayed fully distinct and different activities across our MM cell lines, we then focused on the downstream-located mTOR signaling. To shed light on the molecular mechanism of the mTOR inhibition in MM cell lines, we first analyzed the phosphorylation of the mTOR downstream molecules in temsirolimus-sensitive and -resistant cell lines after 2 and 48 h of temsirolimus treatment. Inhibition of mTOR robustly decreased p-P70S6K and p-S6 levels at both time points in temsirolimus-sensitive cell lines OPM-2, RPMI-8226, and IM-9. Whereas the reduction of p-P70S6K and p-S6 induced in temsirolimus-resistant cell lines AMO-1 and SKMM-2 was observable after 2 h, fully restored phosphorylation of P70S6K and S6 was detected after 48 h (Figure 2A and Figure S7).

We also assessed the inhibition upon the phosphorylation of PI3K downstream proteins after treatment with the pan-PI3K inhibitor pictinisib in our MM cell lines. The phosphorylation of AKT (S473 and T308), PRAS40, P70S6K, and S6 was partially downregulated in a pictinisib-sensitive cell line, MM.1S, and the moderately sensitive cell lines OPM-2, KJON, and OH-2 after pictinisib treatment, but not in the pictinisib-resistant cell lines IM-9 and AMO-1. We also noticed that p-S6 was only reduced in the pictinisib-sensitive cell line, MM.1S, and one pictinisib-moderately sensitive cell line, KJON (Figure S2). These results reaffirm that the pan-class I PI3K inhibitor is poorly effective in our MM cell lines.

### 3.3. Temsirolimus Induces Cell Cycle Arrest and Reduces Cell Proliferation

As the mTOR pathway is considered to mediate the proliferation of MM cell lines, and temsirolimus induced the broadest anti-proliferation activity in our MM cell lines, we next studied the effect of temsirolimus on the survival of MM cell lines. The same panel of cell lines was treated with 10 nM temsirolimus or the corresponding amount of DMSO for 24 h and 48 h prior to the measurement of cell cycle phases and apoptotic rates. To assess proliferation rates, we measured the dilution of CFSE on day 5. Significant G_0_/G_1_ arrest to a different degree was observed in all temsirolimus-sensitive MM cell lines. Most of the temsirolimus-resistant cell lines did not exhibit such changes, except for LP-1 (Figure 2B and Figure S3A). Western blotting analysis of key molecules regulating the G0/G1 phase further supported the observations from cell cycle analysis when cell lines were treated with temsirolimus for 24 h. These included down-modulation of cyclin A, cyclin D1, CDK4, and CDK6 in the temsirolimus-sensitive cell lines OPM-2, KJON, and MM.1S. In contrast, the temsirolimus-resistant cell line SKMM-2 did not show these changes (Figure 2C and Figure S8). Only one temsirolimus-sensitive cell line, KJON, revealed an elevated apoptotic rate upon the treatment with temsirolimus (Figure S3B). In addition, the temsirolimus-sensitive cell lines OPM-2, MM.1S, and KJON showed inhibited proliferation after exposure to temsirolimus (Figure 2D). Thus, we propose that the inhibitory effect of temsirolimus in MM cell lines could be predominantly driven by cell cycle arrest. These results suggest that treatment with temsirolimus alone might be effective in MM.

### 3.4. Combined mTOR and MEK Inhibition Is Synergistic in MM

**Figure 3:**
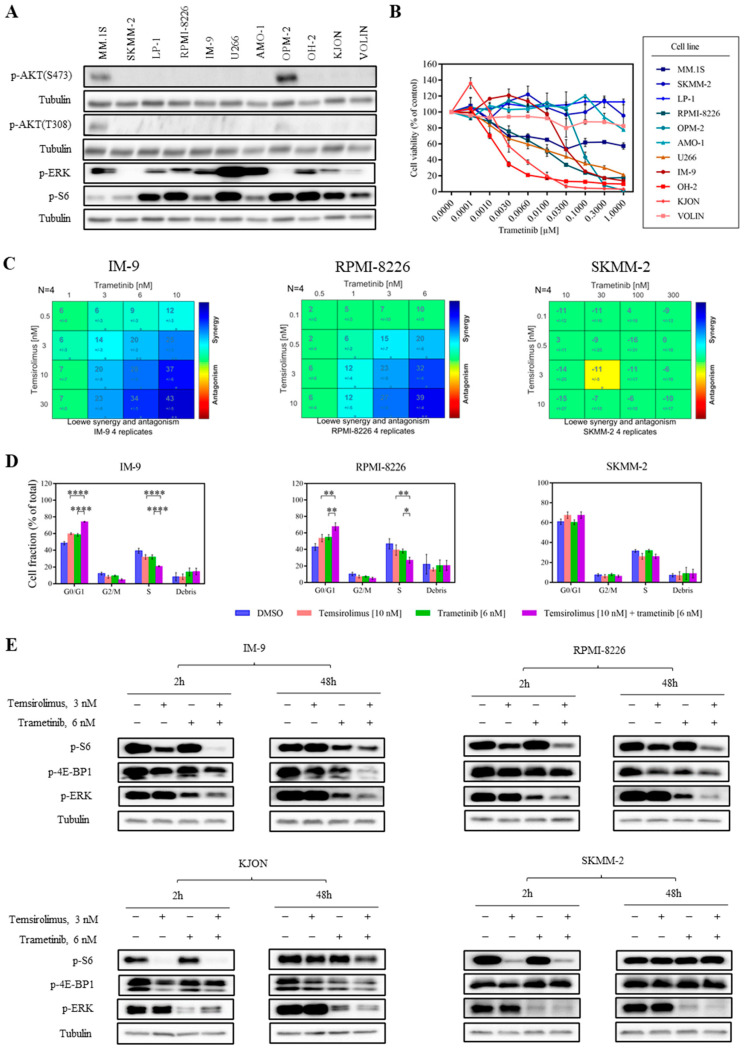
Temsirolimus and trametinib act synergistically in MM cell lines. (**A**) Basal levels of AKT (S473 and T308), ERK, and S6 phosphorylation in MM cell lines were detected by Western blotting using specific antibodies. Tubulin was the loading control. Representative results from at least three independent experiments are shown. (**B**) MM cell lines were incubated for 5 days with MEK inhibitor trametinib. Viable cells were determined. Representative results from at least three independent experiments is shown. Error bars indicate standard deviations. (**C**) Synergy score matrix for temsirolimus and trametinib combination treatment of MM cell lines. Combinatorial treatment induced synergistic cytotoxicity in IM-9 and RPMI-8226 cell lines. In contrast, the SKMM-2 cell line was affected by neither single nor combined inhibitor treatments. (**D**) Cell cycle following treatment with either vehicle control, temsirolimus alone, trametinib alone, or with both temsirolimus and trametinib. Combined inhibitor treatment with temsirolimus and trametinib induced an increase of G_0_/G_1_ stage in IM-9 and RPMI-8226 cell lines compared with either temsirolimus or trametinib alone. In contrast, the SKMM-2 cell line was affected by neither single nor combined inhibitor treatments. Error bars indicate standard deviations. * *p* ≤ 0.05, ** *p* ≤ 0.01, **** *p* ≤ 0.0001. (**E**) Western blotting following treatment with either vehicle control, temsirolimus alone, trametinib alone, or with both temsirolimus and trametinib. Combined inhibitor treatment with temsirolimus and trametinib resulted in a decrease of S6 and ERK phosphorylation in the IM-9 and RPMI-8226 cell lines compared with either temsirolimus or trametinib alone after 2 and 48 h. Combined inhibitor treatment with temsirolimus and trametinib induced a reduction of 4E-BP1 phosphorylation in the IM-9 cell line compared with either temsirolimus or trametinib alone after 48 h. A restoration of S6 phosphorylation was observed in the SKMM-2 cell line following treatment with either temsirolimus alone, or with both temsirolimus and trametinib after 48 h. Tubulin was the loading control. Representative results of three independent experiments are shown.

We then evaluated whether inhibiting another pathway with mTOR would be a promising approach. As a single agent, temsirolimus showed good efficacy in most of the MM cell lines but poor activity in clinical settings, as described above. In line with this, some MM cell lines still exhibited poor responses to temsirolimus alone. Here, we found that p-S6 was detectable in most MM cell lines, whereas AKT phosphorylation (S473 and T308) was only visible in two cell lines (Figure 3A and Figure S9). These data suggest that another upstream regulator of S6 might be responsible for the widely presented p-S6. Using immunoblotting, we observed that phosphorylation of ERK, an indicator of the activated MAPK/ERK pathway, was present in nine of eleven MM cell lines (Figure 3A and Figure S9), primarily implying that the MAPK/ERK pathway was activated in our MM cell lines. The expression of p-ERK in most of our cell lines was positively correlated with p-S6, suggesting crosstalk between the PI3K and MAPK pathways. To dissect if crosstalk between the two pathways exists, we continued further experiments with an inhibitor targeting the MAPK/ERK pathway. Trametinib [46], a MEK inhibitor, showed toxicity in seven of eleven MM cell lines, supporting the activation and the pro-survival role of the MAPK/ERK pathway in these MM cell lines (Figure 3B, Table S2). Subsequently, we performed the combination treatment of temsirolimus and trametinib in 10 MM cell lines by a cell viability assay. The combination of temsirolimus and trametinib was highly synergistic in temsirolimus-sensitive cell lines IM-9 (score range: 6–43), RPMI-8226 (score range: 2–39), and KJON (score range: −3–21) (Figure 3C and Figure S4). Temsirolimus and trametinib combinations displayed an additive or no effect in other MM cell lines (Figure 3C and Figure S4A,B). The Loewe synergy score of each dose combination calculated for each cell line is depicted in Figure 3C and Figure S4B. These results also suggest that there was no correlation between the molecular subtypes of the tested MM cell lines and responses to combination treatment.

As the combination of temsirolimus with trametinib in the cell viability assay revealed synergistic effects, we proceeded with cell cycle and apoptosis studies to elucidate the effects of this combination on the proliferation of the MM cell lines. Based on cell viability assay results, we treated MM cell lines with 10 nM temsirolimus in combination with 6 nM trametinib and examined cell cycle changes after 24 h and apoptosis rates after 48 h. The combination treatment of temsirolimus with trametinib revealed stronger G_0_/G_1_ arrest in IM-9 and RPMI-8226 cell lines. In SKMM-2, the cell line showed no synergy and no increase in the G_0_/G_1_ arrest was detected in combination treatment compared to mono-treatment (Figure 3D). However, we did not observe a significant increase in apoptotic effects in either synergistic or non-synergistic cell lines following single or combination treatments (Figure S4C). These results suggest that the combinatorial effect of temsirolimus and trametinib in MM cell lines is mainly caused by cell cycle inhibition.

To evaluate the molecular mechanism of the combined temsirolimus and trametinib treatment in MM cell lines, we analyzed models that show synergy and no synergy following a 2 and 48 h treatment with either DMSO, temsirolimus, trametinib, or both simultaneously. Co-inhibition decreased the phosphorylation of S6 and ERK in RPMI-8226 and IM-9, two cell lines that showed synergy at both time points compared with either compound alone (Figure 3E and Figure S10). In addition, we could also observe the downregulation of p-4E-BP1 in IM-9 and the decrease of p-S6 and p-ERK in KJON, another synergistic model, were also observed after 48 h treatment (Figure 3E and Figure S10). In contrast, even though the reduction of p-S6 by single temsirolimus or co-inhibition was detected after 2 h in SKMM-2, the cell line showed no synergy, and levels of p-S6 were fully restored after mono- or co-treatment for 48 h (Figure 3E and Figure S10). This could explain why the combined treatment failed to induce an improved anti-proliferation effect in SKMM-2.

Moreover, we also found that the simultaneous inhibition of temsirolimus and trametinib reduced the level of G_0_/G_1_ phase-regulated protein cyclin A in IM-9 and RPMI-8226, two cell lines that showed synergy after 24 h compared with mono-treatment (Figure S4D). Conversely, the level of cyclin A in SKMM-2 did not change after co-inhibition compared with either drug alone (Figure S4D). This also supported the observation from cell cycle analysis. Additionally, to elucidate if co-inhibition also impacts autophagy and ferroptosis, the autophagy markers LC3B-I and LC3B-II [47] were detected by Western blotting, and the anti-ferroptosis rescue assay using a ferroptosis inhibitor was performed. The results revealed that the combined treatment of temsirolimus and trametinib did not lead to an increased level of LC3B-II in any cell lines after 24 h compared to each agent alone (Figure S4D). The addition of the ferroptosis inhibitor ferrostatin-1 [48] failed to restore the cell viability of IM-9 and RPMI-8226 following 5 days of combined treatment (Figure S4E).

## 4. Discussion

This study revealed that the mTOR inhibitor temsirolimus exhibited antiproliferative activity across our MM cell lines, irrespective of the associated genetic changes. Targeting mTOR alone impacted MM proliferation and survival. Moreover, the combination treatment of the mTOR inhibitor with a MEK inhibitor led to a synergistic effect in cell viability and enhanced anti-proliferation efficacy in MM cell lines.

MM can be classified into two distinct genetic subgroups: hyperdiploid (HRD) and nonhyperdiploid (NHRD) [49]. Although almost half of MM patients belong to HRD, very limited studies have been reported on this subgroup. Most of the previous research focused on MM cell lines derived from NHRD patients [50,51,52]. Thus, a broader preclinical study on MM cell lines representing both prognosis types is required. In our study, three of our MM cell lines, OH-2, KJON, and Volin, shared an HRD genotype [34,35], and seven of them harbor IgH translocations that belong to the NHRD genotype. Thus, MM cell lines representing both HRD and NHRD were included. Moreover, our study demonstrated that mTOR inhibition exhibited anti-MM activity, regardless of different genetic aberrations. To a certain extent, our group has filled some gaps in MM research and improved the understanding of MM cell biology. Even though these cell lines have limitations in fully representing different MM subgroups, our study builds the basis for in vivo studies and using primary patient materials in the future.

We observed that phosphorylation of the PI3K key downstream molecule, AKT, was found in only two MM cell lines, whereas further downstream, mTOR, PRAS40, S6, and 4E-BP1 phosphorylation, and the PI3K pathway suppressor, PTEN, were detectable in most MM cell lines (Figure 1B and Figure S6). These results indicate that PI3K might not be the only upstream signal activating mTOR in these cell lines. In our MM panel, only MM.1S, a cell line with detectable p-AKT, showed sensitivity to AKT and some of the screened PI3K inhibitors (Figure 1C and Figure S1). This suggests that the response to PI3K/AKT inhibition in our MM cell lines is positively correlated with the p-AKT level, as expected. According to previous studies, the response to mTOR inhibition has been positively correlated to a high level of AKT phosphorylation [53]. However, in this report, sensitivity to temsirolimus was not positively correlated with the p-AKT level, as the p-AKT was absent in the temsirolimus-sensitive cell lines RPMI-8226, U266, IM-9, KJON, and OH-2 (Figure 1B,D). These data suggest that the status of p-AKT is not a sufficient indicator to predict the sensitivity to mTOR inhibitors in our study, which might be an interesting point to further investigate.

Activated mTORC1 phosphorylates P70S6K, which in turn phosphorylates S6. P-P70S6K and p-S6 positively modulate the protein synthesis that drives cell survival and are commonly used as indicators for mTORC1 activity [54]. To better understand the therapeutic potential of mTOR inhibition in MM, we first investigated the effect of temsirolimus alone in our panel of MM cell lines. The data of our study revealed that temsirolimus blocked mTORC1 activity in the MM cell lines, regardless of their sensitivity after 2 h (Figure 2A and Figure S7). However, after a long incubation time with temsirolimus, this inhibition was not observed in the temsirolimus-resistant cell lines AMO-1 and SKMM-2 but only in temsirolimus-sensitive cell lines OPM-2, RPMI-8226, and IM-9. The results could well explain why these MM cell lines have different responses to temsirolimus, which suggests combining temsirolimus with drugs targeting other pathways to achieve better efficacy. In previous studies, inhibition of mTOR activity could induce cell cycle arrest in the late G1 phase, as it is considered that mTOR plays a role as the final arbiter for nutrient sufficiency before genome replication [55]. This corresponds to our results, which showed a G_0_/G_1_ phase arrestment in eight MM cell lines after temsirolimus treatment (Figure 2B and Figure S3A). Moreover, we noticed this cell cycle arrest in most of our MM cell lines was correlated with the cell viability data, except for one cell line, LP-1, which was resistant to temsirolimus. From previous research, mTORC1 blocks the nuclear function of p27Kip1 as a CDK inhibitor, thus promoting DNA replication and cyclin D–CDK4/6 complex stabilization [56]. Indeed, our study shows reduced levels of cyclin D1, CDK4, and CDK6 in our MM cell lines after temsirolimus treatment (Figure 2C and Figure S8). Additionally, we observed that the inhibition of proliferation in MM cell lines induced by temsirolimus showed consistent results with cell cycle analysis, further supporting that temsirolimus is effective in G_0_/G_1_ phase blockage.

As previously shown, we observed that p-S6 was detected in most of the cell lines, whereas p-AKT was only detected in MM.1S and OPM-2 cell lines (Figure 1B and Figure S6). We speculate that the phosphorylation of S6 in some of our MM panels might have occurred through the activation of other pathways. Previous studies revealed that the MAPK/ERK pathway has crosstalk with the PI3K pathway, as the downstream effector of the MAPK/ERK pathway, ribosomal s6 kinase (RSK), and P70S6K, could regulate different motifs in the same substrate, such as S6 [57]. From immunoblotting results, we found that p-ERK, the indicator of MAPK/ERK signaling activation, was visible in most MM cell lines. Interestingly, the presence of p-ERK was positively correlated with p-S6 in most of our MM cell lines (Figure 3A and Figure S9). Thus, we hypothesize that the phosphorylation of S6 in our MM panel might also be led by the activated MAPK/ERK pathway. Moreover, the positive correlation between the sensitivity to trametinib and the p-S6 level in seven MM cell lines also implies the crosstalk between the MAPK/ERK and PI3K pathways in our MM cell lines (Figure 3B). Therefore, combining an mTOR inhibitor with MEK inhibitors may enhance the anti-MM activity by inhibiting two signaling cascades. In this work, we observed a robust synergistic effect of temsirolimus with the MEK inhibitor trametinib in three MM cell lines (Figure 3C and Figure S4A,B). In the models that showed synergy, the combination treatment with temsirolimus and trametinib reduced S6 phosphorylation to a greater extent than each drug alone (Figure 3E and Figure S10). This finding implies that simultaneous MEK inhibition compensates for the restoration of p-S6 due to the long-term inhibition of mTOR, which might be an attractive point to further investigate. Furthermore, the combination treatment with temsirolimus and trametinib induced a stronger effect on the cell cycle but not apoptosis, autophagy, and ferroptosis (Figure 3D and Figure S4C–E); meanwhile, the G_0_/G_1_ phase-regulated protein cyclin A was decreased to a greater extent after co-inhibition (Figure S4D). These observations suggested that the synergism was mainly led by cell cycle arrest.

Interestingly, we also observed that p-ERK or p-4E-BP1 were also downregulated to a greater extent in our MM cell lines after the combination treatment of temsirolimus and trametinib compared to each agent alone (Figure 3E and Figure S10); this needs to be interpreted by further experiments. Moreover, as Uddin Md. Nazim et al. [58] described the combination treatment of the tyrosine kinase inhibitor ponatinib and the mTORC1 inhibitor sirolimus significantly reduced xenograft MM model tumor growth and weight compared to mono-treatment without obvious toxicity induction such as liver and kidney function damage or bodyweight loss. In a phase I clinical trial of relapsed or refractory MM, the combination of the mTOR inhibitor everolimus and the immunomodulatory agent lenalidomide impressively prolonged the median overall survival of patients [59]. This combination was also well tolerated and exhibited good responses in a heavily pretreated group. Thus, the efficacy displayed by these previous combinations of mTOR inhibitors with other drugs, such as kinase inhibitors or classical agents, also suggests the future availability of our combinations for in vivo or clinical evaluation. In addition, temsirolimus with trametinib could be combined at achievable and low doses, making this combination attractive in the clinic. Our data also support the hypothesis that mTOR inhibitors can maximize benefits when combined with other agents rationally.

## 5. Conclusions

Taken together, our data show that the combination of mTOR with MEK inhibitors could provide some rationale to test this in further in vivo studies and potentially in a clinical setting. It suggests that MM cases with high S6 phosphorylation but low AKT phosphorylation could benefit from this combination. This is independent of cytogenetic findings and unveils an additional way to classify MM cells. In summary, these results indicate that the effect of temsirolimus alone in MM could be enhanced by combination treatment with MEK inhibition.

## Data Availability

The data presented in this study are openly available.

## Supplementary Materials

The following supporting information can be downloaded at: https://www.mdpi.com/article/10.3390/cancers15082373/s1, Figure S1: Different sensitivities of the MM cell lines to the PI3K isoforms and AKT inhibitors; Figure S2: Pictinisib is poorly active in vitro; Figure S3: mTOR inhibitor treatments in MM cell lines; Figure S4: Temsirolimus and trametinib act synergistically in MM cell lines; Figure S5: Original Western blots for Figure 1A; Figure S6: Original Western blots for Figure 1B; Figure S7: Original Western blots for Figure 2A; Figure S8: Original Western blots for Figure 2C; Figure S9: Original Western blots for Figure 3A; Figure S10: Original Western Blots for Figure 3E.; Table S1: MM cell lines based on cytogenetics; Table S2: IC50 values of 11 MM cell lines for pictilisib, temsirolimus, and trametinib.
