# Supplementary material for: Combination Treatment Targeting mTOR and MAPK Pathways Has Synergistic Activity in Multiple Myeloma
Source: Cancers (Basel). 2023 Apr 19;15(8):2373. doi: 10.3390/cancers15082373 (PMC10136620; doi:10.3390/cancers15082373)
Supplement: Supplementary file 1 [file cancers-15-02373-s001.zip › cancers-2245725-Supplemental figures and tables - revised.pdf]

Figure S1.

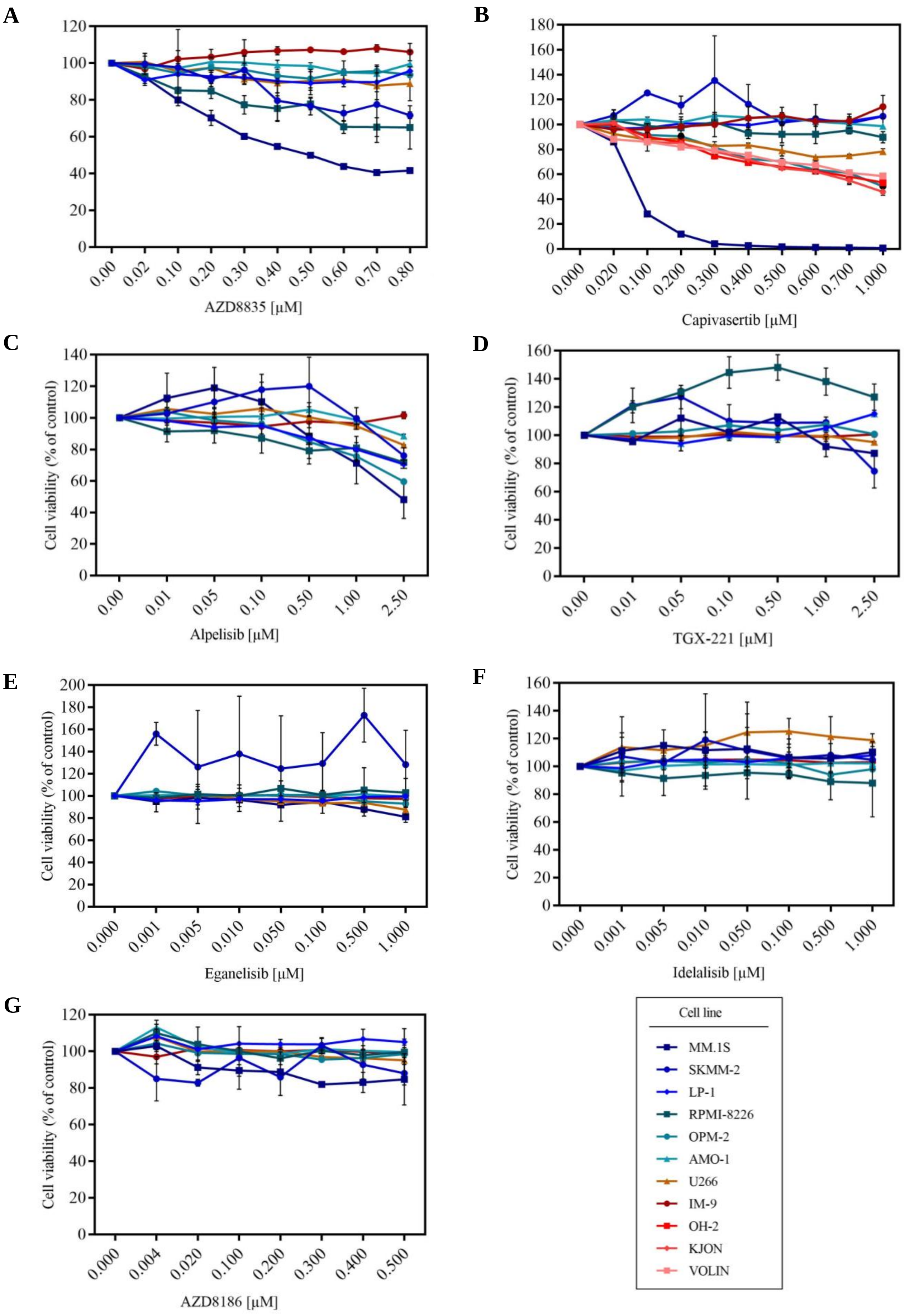
A
120
100
80
60
40
20
0
0.00
0.02
0.10
0.20
0.30
0.40
0.50
0.60
0.70
0.80
AZD8835 [μM]
B
180
160
140
120
100
80
60
40
20
0
0.000
0.020
0.100
0.200
0.300
0.400
0.500
0.600
0.700
1.000
Capivasertib [μM]
C
Cell viability (% of control)
140
120
100
80
60
40
20
0
0.00
0.01
0.05
0.10
0.50
1.00
2.50
Alpelisib [μM]
D
Cell viability (% of control)
160
140
120
100
80
60
40
20
0
0.00
0.01
0.05
0.10
0.50
1.00
2.50
TGX-221 [μM]
E
Cell viability (% of control)
200
180
160
140
120
100
80
60
40
20
0
0.000
0.001
0.005
0.010
0.050
0.100
0.500
1.000
Eganelisib [μM]
F
Cell viability (% of control)
160
140
120
100
80
60
40
20
0
0.000
0.001
0.005
0.010
0.050
0.100
0.500
1.000
Idelalisib [μM]
G
Cell viability (% of control)
120
100
80
60
40
20
0
0.000
0.004
0.020
0.100
0.200
0.300
0.400
0.500
AZD8186 [μM]
Cell line
MM.1S
SKMM-2
LP-1
RPMI-8226
OPM-2
AMO-1
U266
IM-9
OH-2
KJON
VOLIN

Figure S1. Different sensitivities of MM cell lines to PI3K isoforms and AKT inhibitors **(A)** MM cell lines were incubated for 5 days with PI3Kα/δ inhibitor AZD8835. Viable cells were determined. Representative result from at least 3 independent experiments is shown. Error bars indicate standard deviations. **(B)** MM cell lines were incubated for 5 days with AKT inhibitor capivasertib. Viable cells were determined. Representative result from at least 3 independent experiments is shown. Error bars indicate standard deviations. **(C)** MM cell lines were incubated for 5 days with PI3Kα inhibitor alpelisib. Viable cells were determined. Representative result from at least 3 independent experiments is shown. Error bars indicate standard deviations. **(D)** MM cell lines were incubated for 5 days with PI3Kβ inhibitor TGX-221. Viable cells were determined. Representative result from at least 3 independent experiments is shown. Error bars indicate standard deviations. **(E)** MM cell lines were incubated for 5 days with PI3Kγ inhibitor eganelisib. Viable cells were determined. Representative result from at least 3 independent experiments is shown. Error bars indicate standard deviations. **(F)** MM cell lines were incubated for 5 days with PI3Kδ inhibitor idelalisib. Viable cells were determined. Representative result from at least 3 independent experiments is shown. Error bars indicate standard deviations. **(G)** MM cell lines were incubated for 5 days with PI3Kβ/δ inhibitor AZD8186. Viable cells were determined. Representative result from at least 3 independent experiments is shown. Error bars indicate standard deviations.

Figure S2.

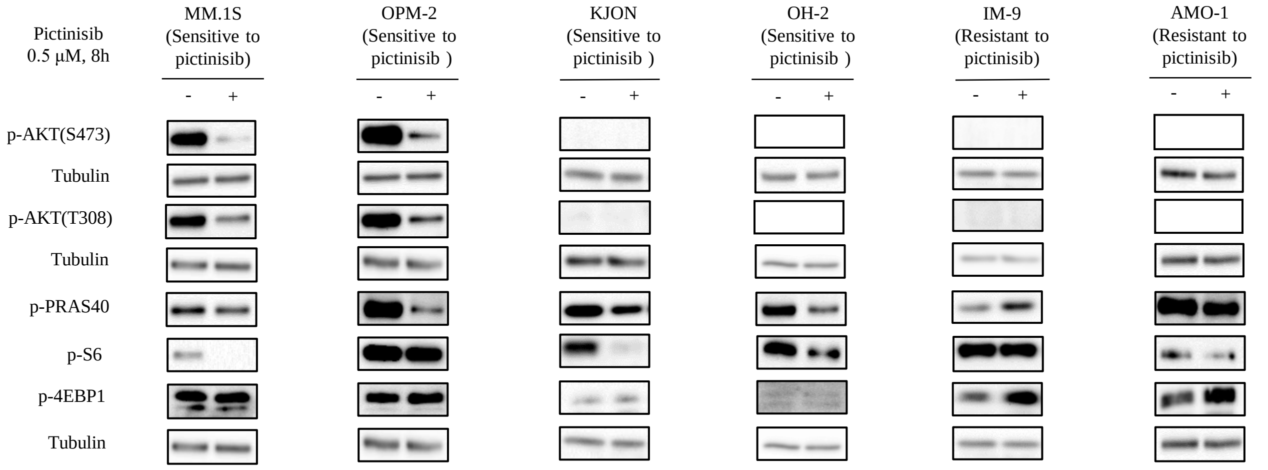

Figure S2. Pictinisib is poorly active *in vitro*. Treatment with 0.5 µM pictinisib for 8 hours resulted in partially decreased phosphorylation of PI3K downstream molecules in MM.1S, OPM-2, KJON, and OH-2 cell lines. No downregulation could be observed in IM-9 and AMO-1 cell lines. Tubulin was the loading control. These results are representative of three separate experiments.

Figure S3.

A

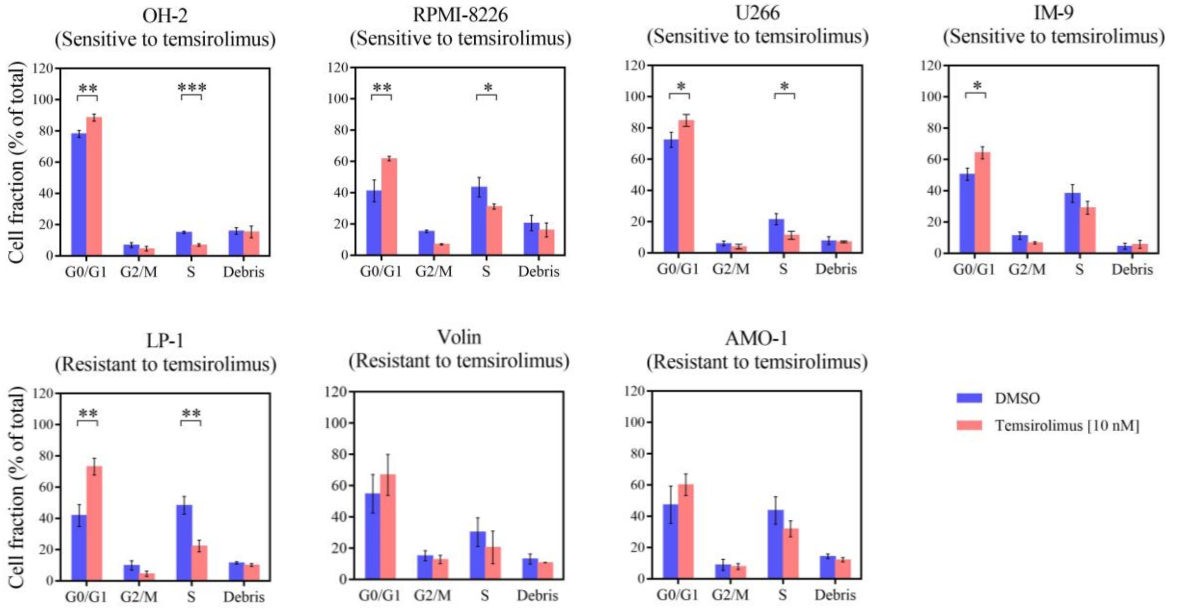
OH-2
(Sensitive to temsirolimus)
RPMI-8226
(Sensitive to temsirolimus)
U266
(Sensitive to temsirolimus)
IM-9
(Sensitive to temsirolimus)
LP-1
(Resistant to temsirolimus)
Volin
(Resistant to temsirolimus)
AMO-1
(Resistant to temsirolimus)
Cell fraction (% of total)
G0/G1
G2/M
S
Debris
DMSO
Temsirolimus [10 nM]

B

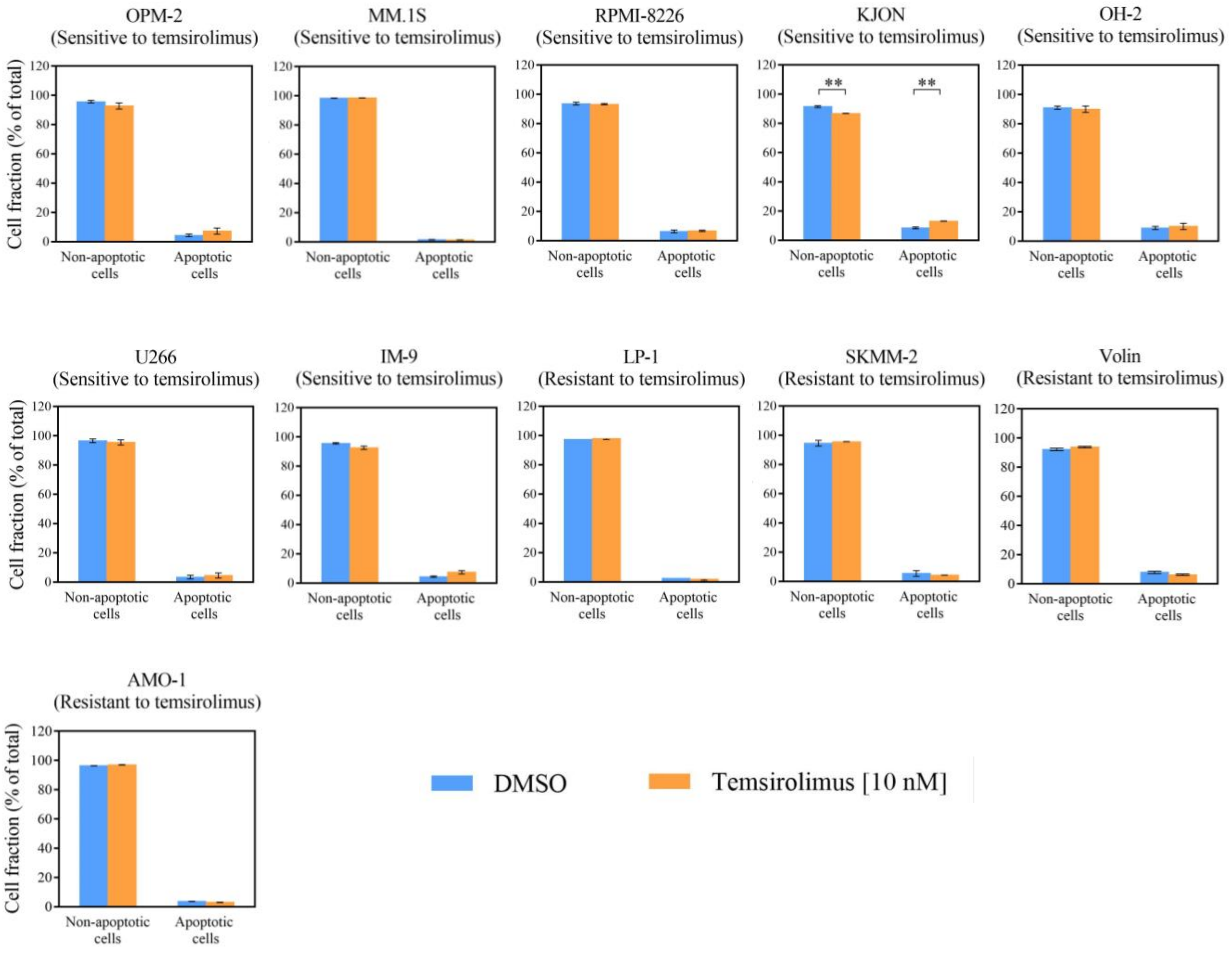
OPM-2
(Sensitive to temsirolimus)
MM.1S
(Sensitive to temsirolimus)
RPMI-8226
(Sensitive to temsirolimus)
KJON
(Sensitive to temsirolimus)
OH-2
(Sensitive to temsirolimus)
U266
(Sensitive to temsirolimus)
IM-9
(Sensitive to temsirolimus)
LP-1
(Resistant to temsirolimus)
SKMM-2
(Resistant to temsirolimus)
Volin
(Resistant to temsirolimus)
AMO-1
(Resistant to temsirolimus)
Cell fraction (% of total)
Non-apoptotic cells
Apoptotic cells
DMSO
Temsirolimus [10 nM]

Figure S3. mTOR inhibitor treatments in MM cell lines **(A)** Temsirolimus treatment induced cell cycle arrest. 24 hours of inhibitor treatment resulted in an increase of G_0/G_1 stage in OH-2, RPMI-8226, U266, LP-1, and IM-9 cell lines. Data are expressed as means ± standard deviation of at least 3 independent experiments. Error bars indicate standard deviations. $P^{*} \leq 0.05$, $P^{**} \leq 0.01$, $P^{***} \leq 0.001$. **(B)** 48 hours of temsirolimus treatment did not induce a significant increase of apoptosis in MM cell lines except for KJON cell line. Representative results of 3 independent experiments are shown. Error bars indicate standard deviations. $P^{**} \leq 0.01$.

Figure S4.

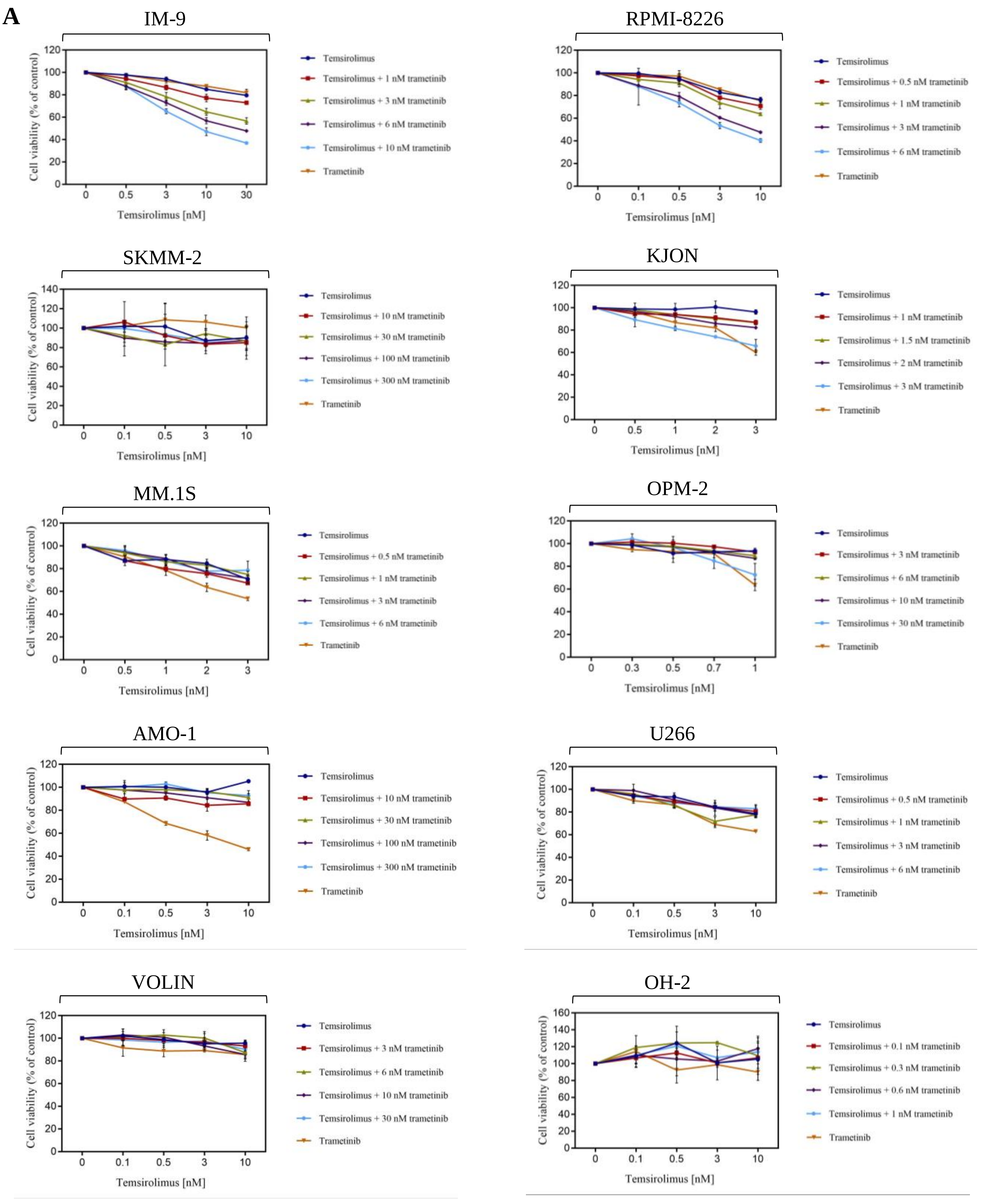
A
IM-9
Cell viability (% of control)
Temsirolimus [nM]
Temsirolimus
Temsirolimus + 1 nM trametinib
Temsirolimus + 3 nM trametinib
Temsirolimus + 6 nM trametinib
Temsirolimus + 10 nM trametinib
Trametinib
RPMI-8226
Cell viability (% of control)
Temsirolimus [nM]
Temsirolimus
Temsirolimus + 0.5 nM trametinib
Temsirolimus + 1 nM trametinib
Temsirolimus + 3 nM trametinib
Temsirolimus + 6 nM trametinib
Trametinib
SKMM-2
Cell viability (% of control)
Temsirolimus [nM]
Temsirolimus
Temsirolimus + 10 nM trametinib
Temsirolimus + 30 nM trametinib
Temsirolimus + 100 nM trametinib
Temsirolimus + 300 nM trametinib
Trametinib
KJON
Temsirolimus [nM]
Temsirolimus
Temsirolimus + 1 nM trametinib
Temsirolimus + 1.5 nM trametinib
Temsirolimus + 2 nM trametinib
Temsirolimus + 3 nM trametinib
Trametinib
MM.1S
Cell viability (% of control)
Temsirolimus [nM]
Temsirolimus
Temsirolimus + 0.5 nM trametinib
Temsirolimus + 1 nM trametinib
Temsirolimus + 3 nM trametinib
Temsirolimus + 6 nM trametinib
Trametinib
OPM-2
Temsirolimus [nM]
Temsirolimus
Temsirolimus + 3 nM trametinib
Temsirolimus + 6 nM trametinib
Temsirolimus + 10 nM trametinib
Temsirolimus + 30 nM trametinib
Trametinib
AMO-1
Cell viability (% of control)
Temsirolimus [nM]
Temsirolimus
Temsirolimus + 10 nM trametinib
Temsirolimus + 30 nM trametinib
Temsirolimus + 100 nM trametinib
Temsirolimus + 300 nM trametinib
Trametinib
U266
Cell viability (% of control)
Temsirolimus [nM]
Temsirolimus
Temsirolimus + 0.5 nM trametinib
Temsirolimus + 1 nM trametinib
Temsirolimus + 3 nM trametinib
Temsirolimus + 6 nM trametinib
Trametinib
VOLIN
Cell viability (% of control)
Temsirolimus [nM]
Temsirolimus
Temsirolimus + 3 nM trametinib
Temsirolimus + 6 nM trametinib
Temsirolimus + 10 nM trametinib
Temsirolimus + 30 nM trametinib
Trametinib
OH-2
Cell viability (% of control)
Temsirolimus [nM]
Temsirolimus
Temsirolimus + 0.1 nM trametinib
Temsirolimus + 0.3 nM trametinib
Temsirolimus + 0.6 nM trametinib
Temsirolimus + 1 nM trametinib
Trametinib

Figure S4.

B

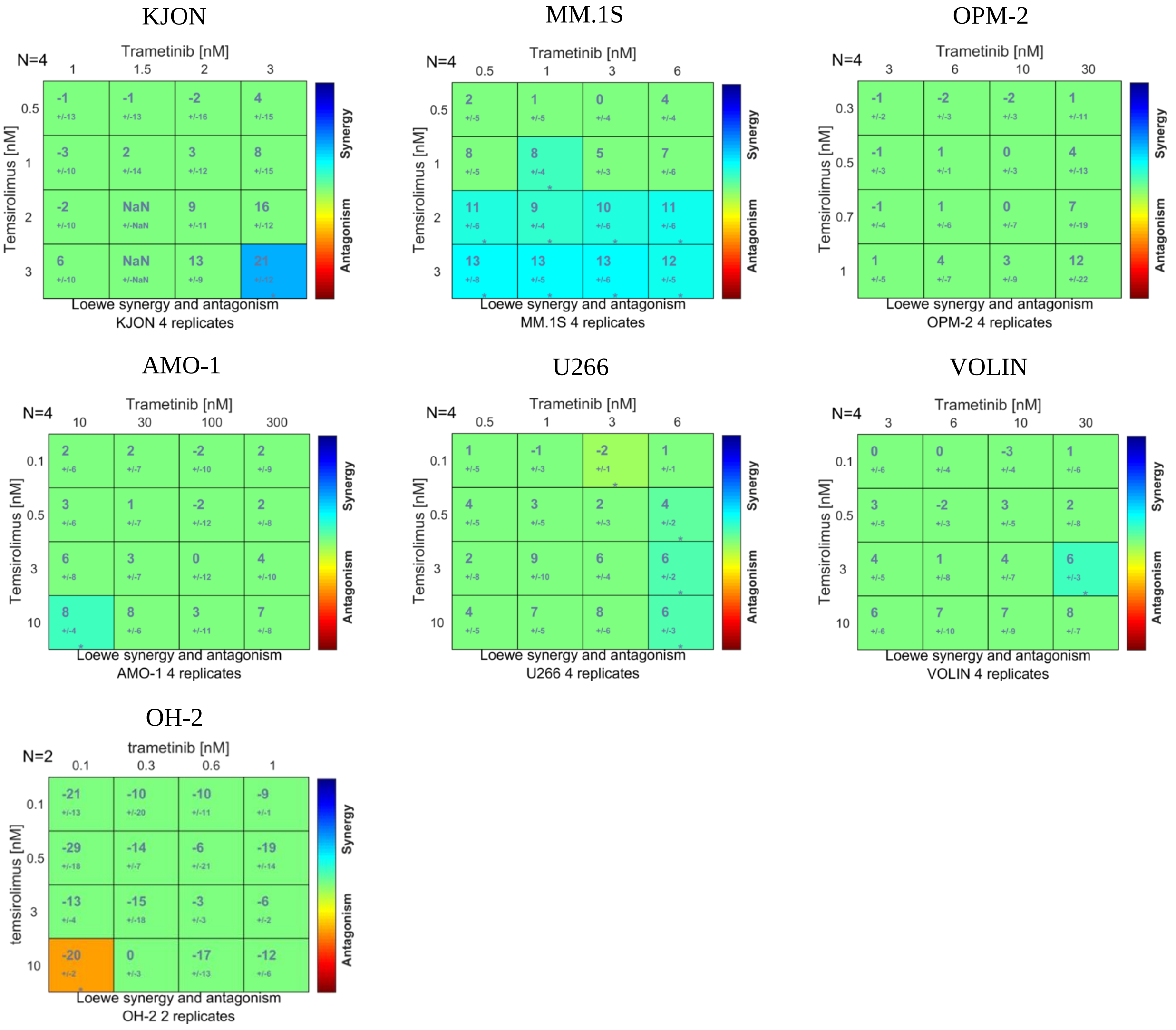

C

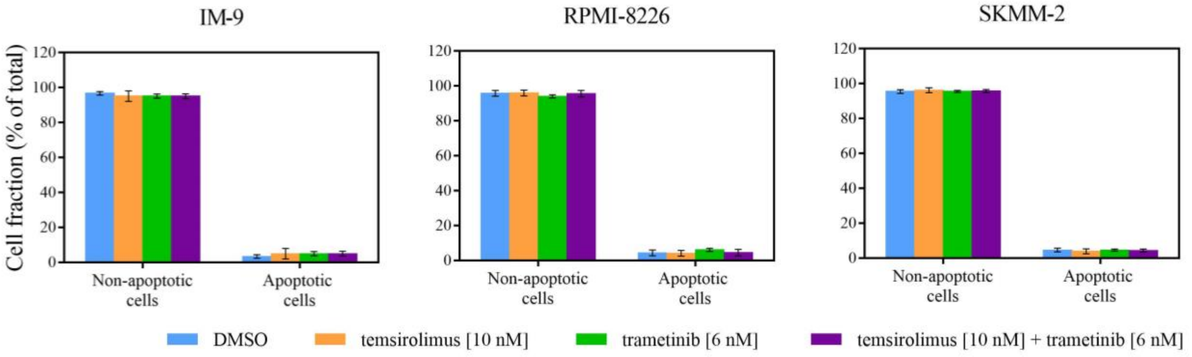

D

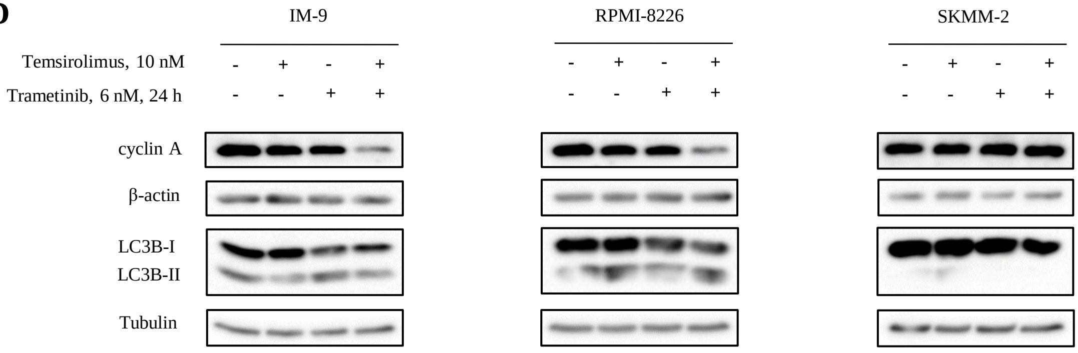

Figure S4.

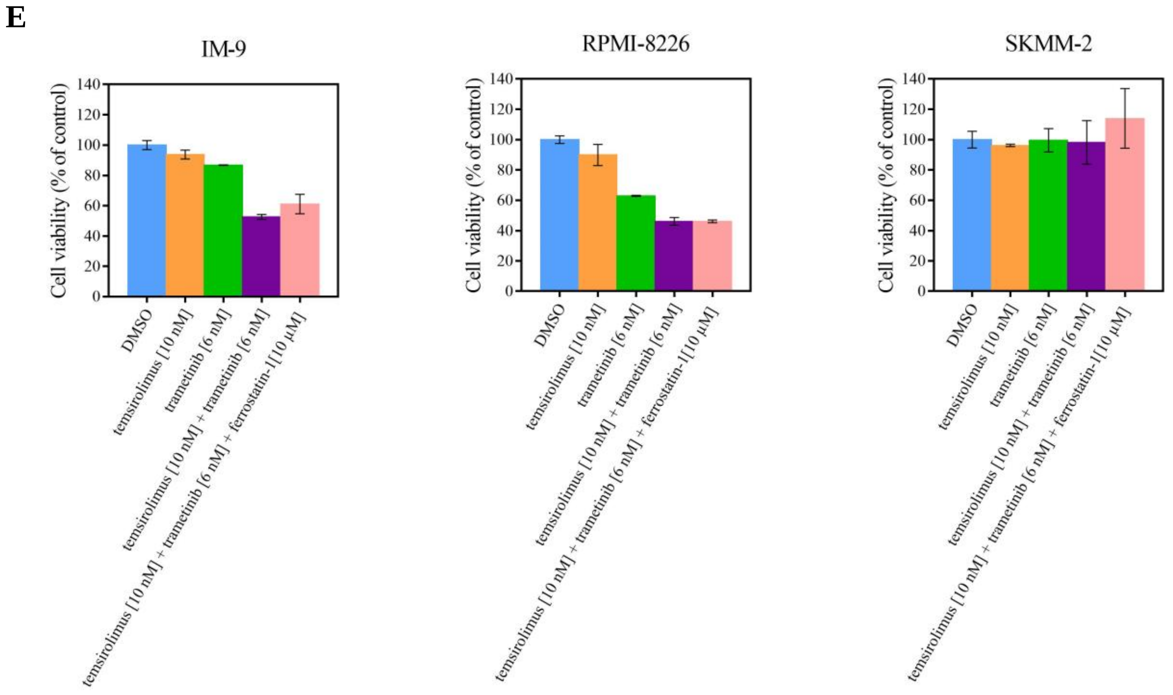

Figure S4. Temsirolimus and trametinib act synergistically in MM cell lines. **(A)** Temsirolimus and trametinib combination treatment in 10 MM cell lines. MM cell lines were incubated for 5 days with mTOR inhibitor temsirolimus and MEK inhibitor trametinib. Viable cells were determined. Representative results from at least 3 independent experiments are shown. Error bars indicate standard deviations. **(B)** Synergy score matrix for temsirolimus and trametinib combination treatment in 7 MM cell lines. **(C)** Apoptosis following treatment with either vehicle control, temsirolimus alone, trametinib alone, or with both temsirolimus and trametinib. Combined inhibitor treatment with temsirolimus and trametinib did not induce an increase of apoptosis in IM-9, RPMI-8226, and SKMM-2 cell lines compared with either temsirolimus or trametinib alone. Representative results of 3 independent experiments are shown. Error bars indicate standard deviations. **(D)** Western Blotting following treatment with either vehicle control, temsirolimus alone, trametinib alone, or with both temsirolimus and trametinib. Combined inhibitor treatment with temsirolimus and trametinib decreased the level of cyclin A in IM-9 and RPMI-8226 cell lines but not in SKMM-2 cell line compared with either temsirolimus or trametinib alone after 24 hours. Combination treatment did not increase the level of LC3B-II in IM-9, RPMI-8226, and SKMM-2 cell lines. β-actin and tubulin were the loading control. Representative results of 3 independent experiments are shown. **(E)** Anti-ferroptosis rescue following treatment with temsirolimus and trametinib. MM cell lines were incubated for 5 days with mTOR inhibitor temsirolimus, MEK inhibitor trametinib, and ferroptosis inhibitor ferrostatin-1. Viable cells were determined. The addition of ferrostatin-1 failed to rescue the survival of IM-9, RPMI-8226, and SKMM-2 cell lines after combination treatment with temsirolimus and trametinib. Representative results of 2 independent experiments are shown.

Figure S5.

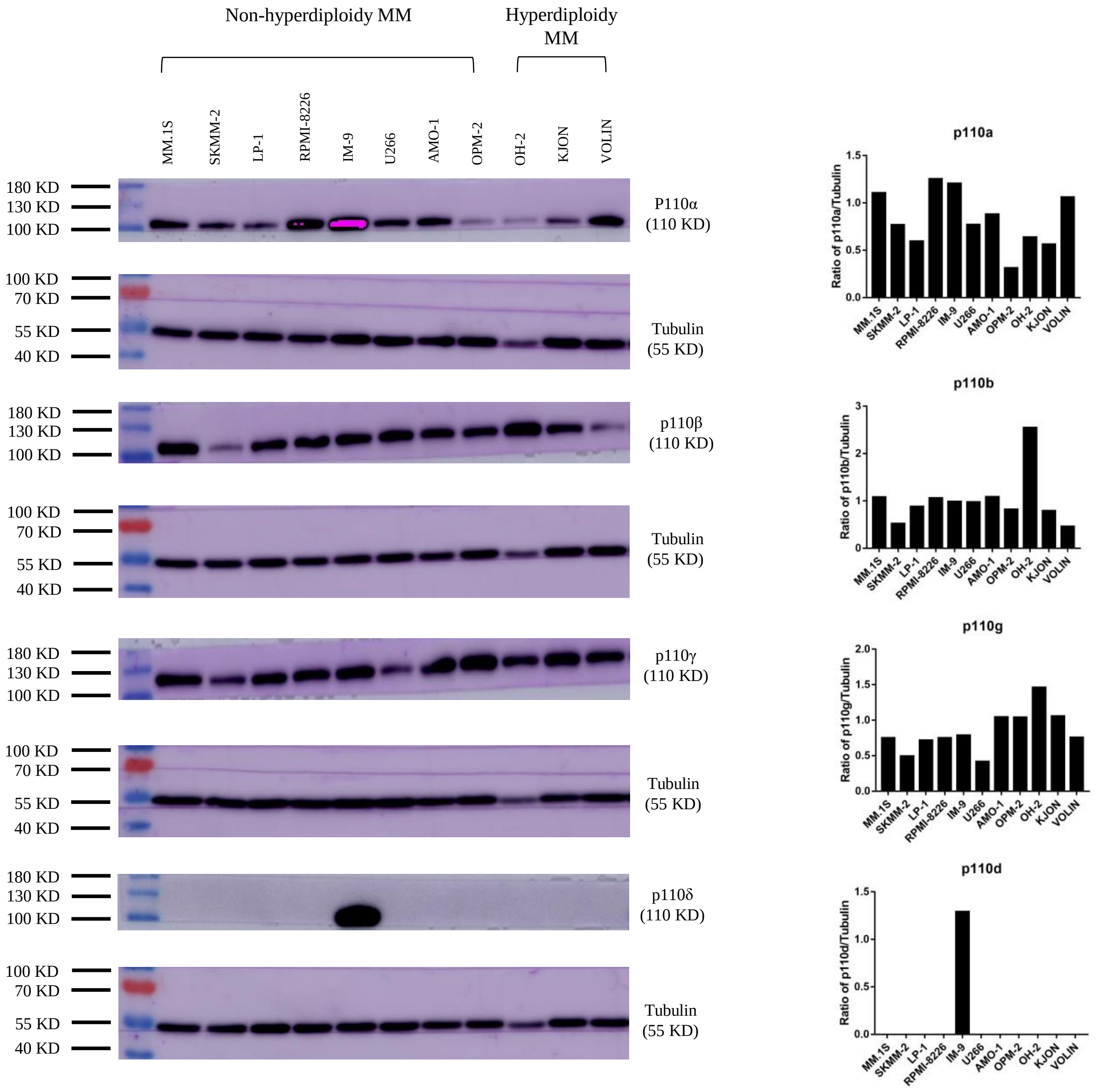

Figure S5. Original Western Blots for Figure 1A. Protein band densitometric analysis was performed by ImageJ software and is shown next to each blot.

Figure S6.

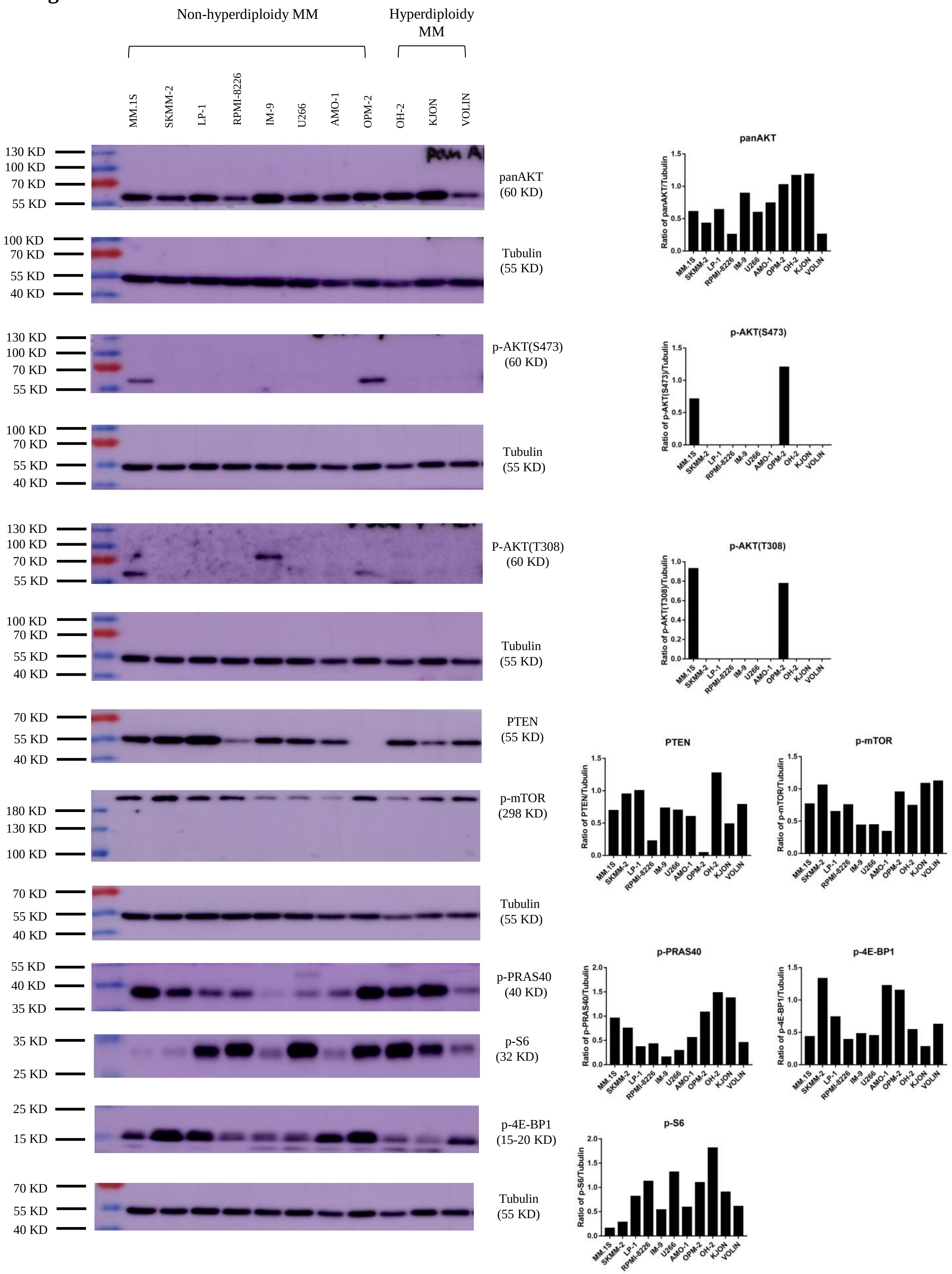

Figure S6. Original Western Blots for Figure 1B. Protein band densitometric analysis was performed by ImageJ software and is shown next to each blot.

Figure S7.

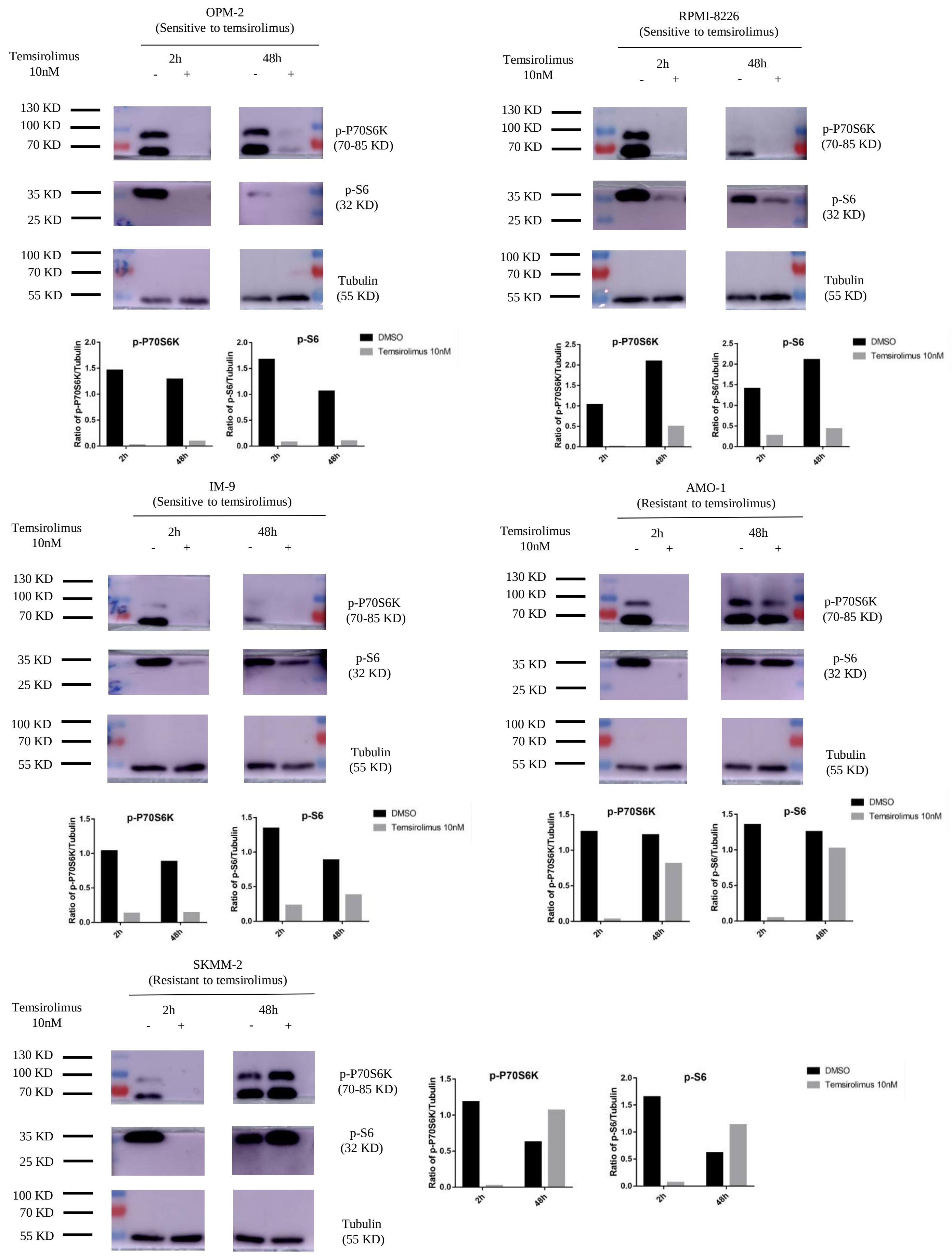

Figure S7. Original Western Blots for Figure 2A. Protein band densitometric analysis was performed by ImageJ software and is shown next to each blot.

Figure S8.

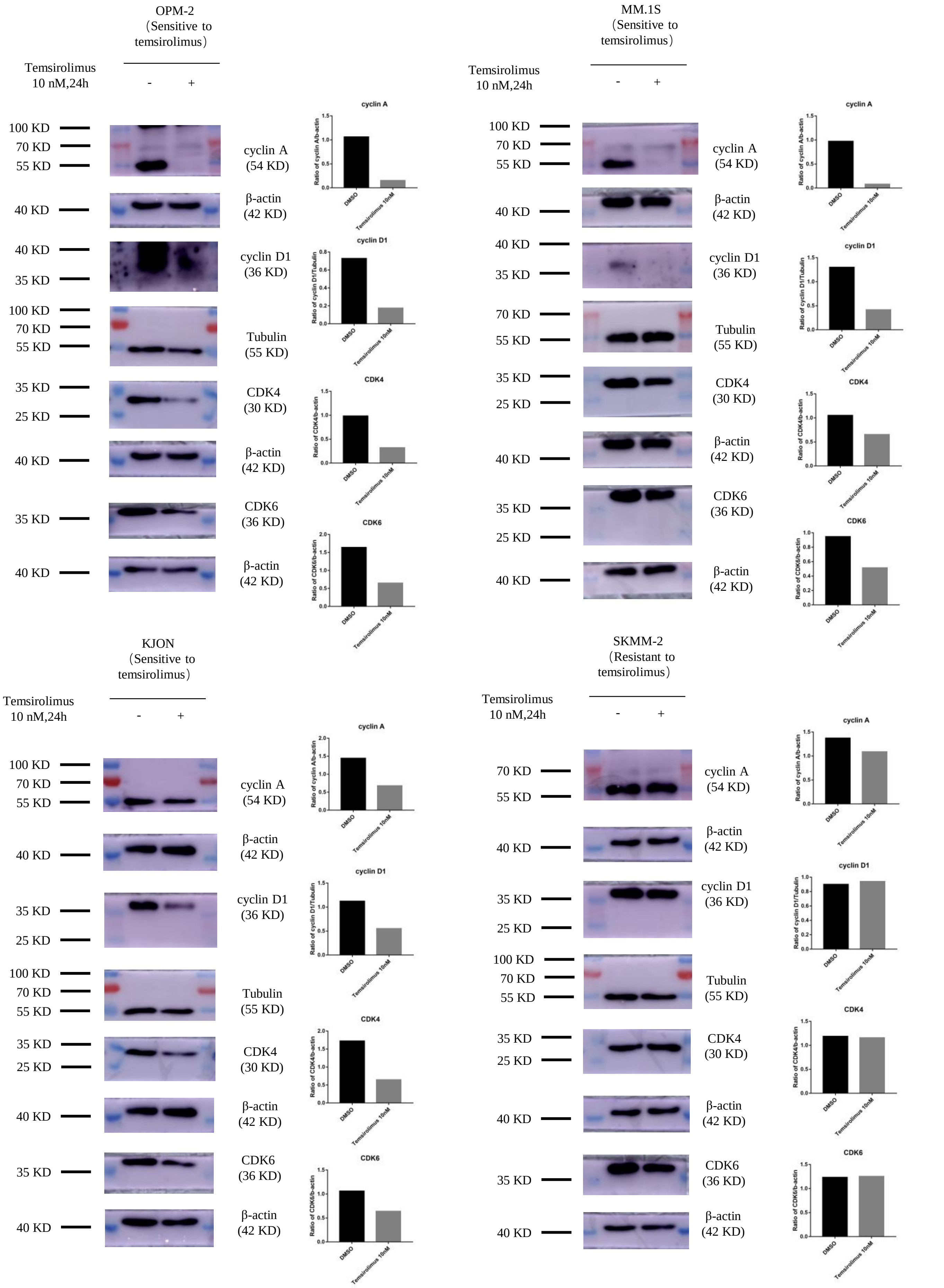

Figure S8. Original Western Blots for Figure 2C. Protein band densitometric analysis was performed by ImageJ software and is shown next to each blot.

Figure S9.

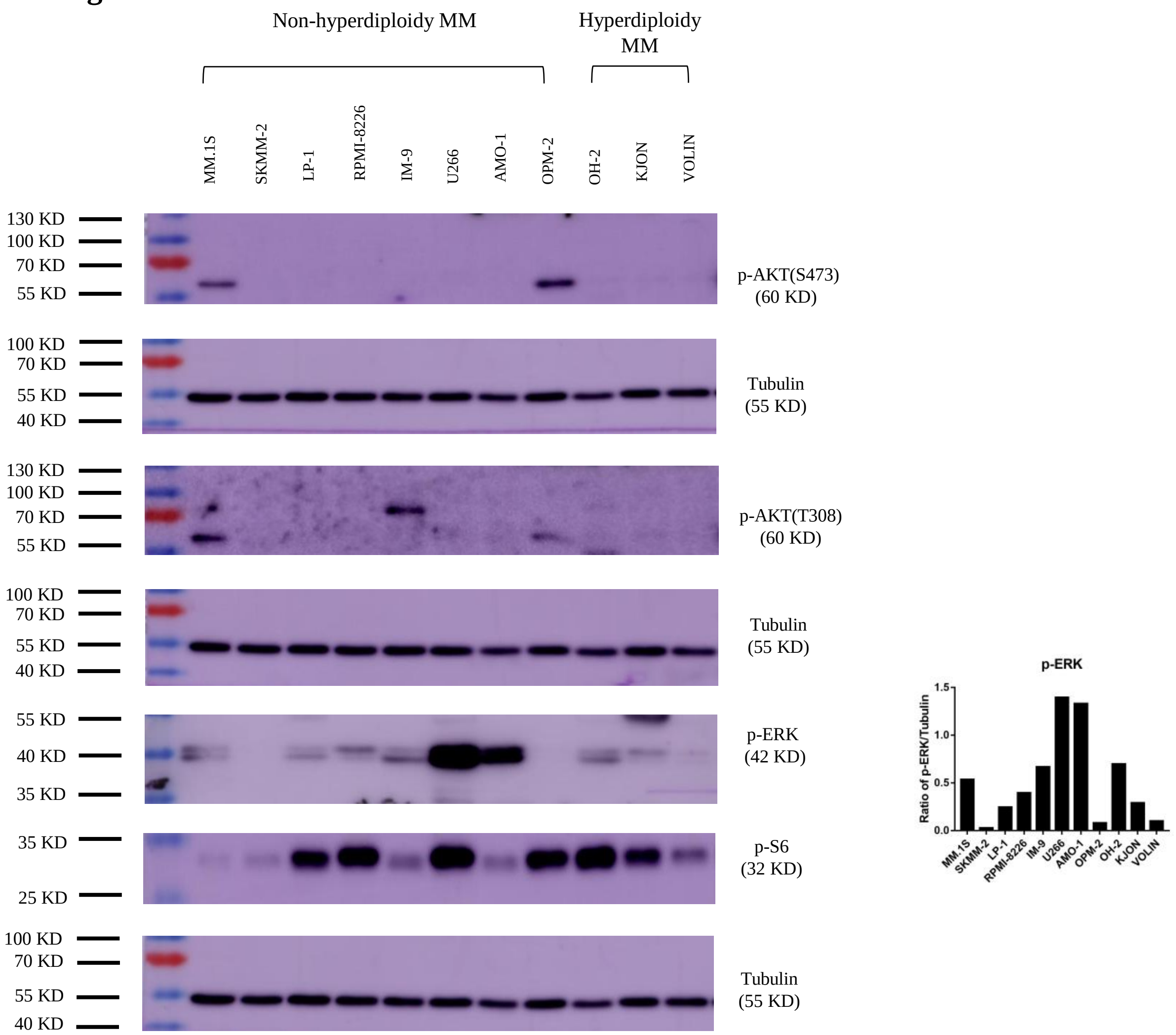

Figure S9. Original Western Blots for Figure 3A. Protein band densitometric analysis was performed by ImageJ software and is shown next to each blot.

Figure S10.

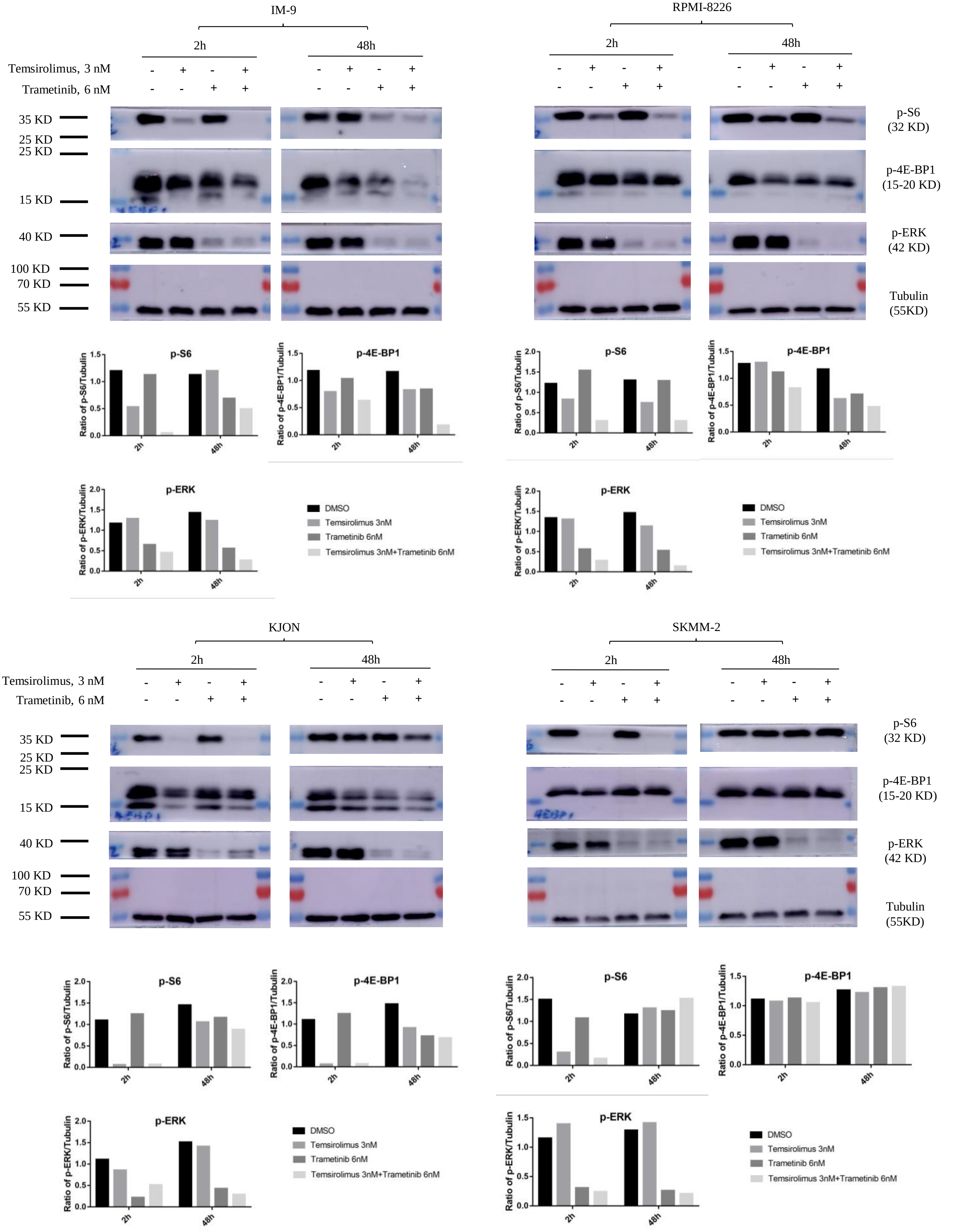

Figure S10. Original Western Blots for Figure 3E. Protein band densitometric analysis was performed by ImageJ software and is shown next to each blot.

Table S1. MM cell lines based on cytogenetics

Cell line	Genetic aberrations	Prognosis of cytogenetic alteration among MM patients
MM.1S	t 14;16 (*c-Maf*) ; 1q21 (*CSK1B*) gain [33]	Poor
SKMM-2	del17p (*TP53*); t 11; 14 (*CCND1*) [33]	Poor
LP-1	t 4;14 (*MMSET/FGFR3*); 1q21 (*CSK1B*) gain [33]	Poor
RPMI-8226	t 14;16 (*c-Maf*) ; 1q21 (*CSK1B*) gain; del17p (*TP53*) [33]	Poor
OPM-2	t 4;14 (*MMSET/FGFR3*); 1q21 (*CSK1B*) gain; del17p (*TP53*) [33]	Poor
AMO-1	1q21 (*CSK1B*) gain; t 12; 14 (*CCND2*) [33]	Poor
U266	t 11; 14 (*CCND1*) [33]	Intermediate
IM-9	B-lymphoblastoid cell line [32]	Good
OH-2	hyperdiploidy chromosomes 3,7,15,19 and 21 [35]	Good
KJON	hyperdiploidy chromosomes 3,5,7,9,11,17 and 21 [34]	Good
VOLIN	hyperdiploidy chromosomes 3,5,7,9,11,17 and 21 [34]	Good

References

32. Pellat-Deceunynck C, Amiot M, Bataille R, van Riet I, van Camp B, Omede P, et al. Human myeloma cell lines as a tool for studying the biology of multiple myeloma: a reappraisal 18 years after. *Blood* **1995**;*86*(10),4001–4002.

33. Moreaux J, Klein B, Bataille R, Descamps G, Maïga S, Hose D, et al. A high-risk signature for patients with multiple myeloma established from the molecular classification of human myeloma cell lines. Haematologica. **2011**;*96*(4), 574.

34. Våtsveen, T.K.; Børset, M.; Dikic, A.; Tian, E.; Micci, F.; Lid, A.H.B.; Meza-Zepeda, L.A.; Coward, E.; Waage, A.; Sundan, A.; et al. VOLIN and KJON—Two novel hyperdiploid myeloma cell lines. *Genes Chromosomes Cancer* **2016**, *55*, 890–901.

35. Våtsveen, T.K.; Tian, E.; Kresse, S.H.; Meza-Zepeda, L.A.; Gabrea, A.; Glebov, O.; Dai, H.Y.; Sundan, A.; Kuehl, W.M.; Børset, M. OH-2, a hyperdiploid myeloma cell line without an IGH translocation, has a complex translocation juxtaposing MYC near MAFB and the IGK locus. *Leuk. Res.* **2009**, *33*, 1670–1677.

Table S2. IC50 values of 11 MM cell lines for pictilisib, temsirolimus, and trametinib

Cell line	Prognosis of cytogenetic alteration among MM patients	IC50 of pictinisib (µM)	IC50 of temsirolimus (µM)	IC50 of trametinib (µM)
MM.1S	Poor	0.51	0.055	0.86
SKMM-2	Poor	1.61	Not available	Not available
LP-1	Poor	5.24	6.29	Not available
RPMI-8226	Poor	16.63	0.081	0.015
OPM-2	Poor	2.21	0.0031	0.093
AMO-1	Poor	Not available	Not available	1.9
U266	Intermediate	4.43	2.38	0.022
IM-9	Good	Not available	1.03	0.045
OH-2	Good	1.4	0.45	0.002
KJON	Good	1.07	0.15	0.0042
VOLIN	Good	1.29	Not available	7905
